# Pulse Dipolar EPR Reveals Double-Histidine Motif Cu^II^–NTA Spin-Labeling Robustness against Competitor Ions

**DOI:** 10.1021/acs.jpclett.1c00211

**Published:** 2021-03-13

**Authors:** Joshua L. Wort, Swati Arya, Katrin Ackermann, Alan J. Stewart, Bela E. Bode

**Affiliations:** ^†^EaStCHEM School of Chemistry, ^‡^Biomedical Sciences Research Complex, Centre of Magnetic Resonance, University of St. Andrews, North Haugh, St. Andrews, KY16 9ST, U.K.; § School of Medicine, University of St. Andrews, North Haugh, St. Andrews, KY16 9TF, U.K.

## Abstract

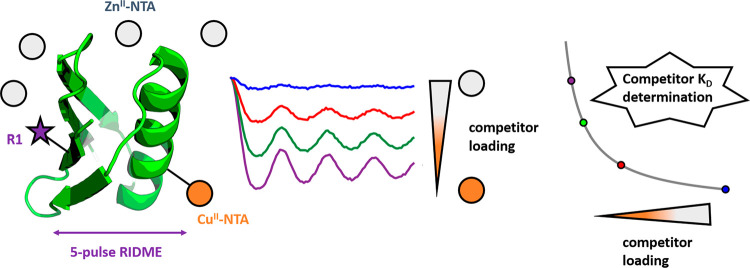

Pulse-dipolar EPR is an appealing strategy for structural characterization of complex systems in solution that complements other biophysical techniques. Significantly, the emergence of genetically encoded self-assembling spin labels exploiting exogenously introduced double-histidine motifs in conjunction with Cu^II^-chelates offers high precision distance determination in systems nonpermissive to thiol-directed spin labeling. However, the noncovalency of this interaction exposes potential vulnerabilities to competition from adventitious divalent metal ions, and pH sensitivity. Herein, a combination of room-temperature isothermal titration calorimetry (ITC) and cryogenic relaxation-induced dipolar modulation enhancement (RIDME) measurements are applied to the model protein *Streptococcus sp.* group G. protein G, B1 domain (GB1). Results demonstrate double-histidine motif spin labeling using Cu^II^-nitrilotriacetic acid (Cu^II^–NTA) is robust against the competitor ligand Zn^II^–NTA at >1000-fold molar excess, and high nM binding affinity is surprisingly retained under acidic and basic conditions even though room temperature affinity shows a stronger pH dependence. This indicates the strategy is well-suited for diverse biological applications, with the requirement of other metal ion cofactors or slightly acidic pH not necessarily being prohibitive.

As the complexity of biomolecular assemblies implicated in health and disease has increased, so too has interest in pulse-dipolar EPR (PD-EPR) as a robust strategy for solution-state structural characterization of proteins^1,2^ and nucleic acids^3,4^ in the nanometer distance regime.^5,6^ PD-EPR is a powerful tool that complements X-ray crystallography, NMR, cryo-EM, and Förster Resonance Energy Transfer (FRET) data by providing structural insight without the need for crystallization, size limitation, or structurally perturbative labels. Hence, PD-EPR has been applied to study conformational equilibria,^7^ oligomerization degree,^8,9^ complexation events,^10−12^ and competing structural models.^13^

Pairs of paramagnetic moieties are commonly introduced into diamagnetic systems of interest using thiol-based site-directed spin labeling.^14^ Cysteine residues are typically covalently modified, as for the nitroxide R1 side chain (Figure 1a top). This strategy is suboptimal in systems containing essential cysteine residues, nonpermissive to post-translational reduction. However, Cu^II^-based genetically encodable self-assembling spin labels using double-histidine motifs have emerged as an alternative labeling strategy.^15,16^ Additionally, the bipedal mode of Cu^II^-chelate attachment at the double-histidine motif (Figure 1a bottom) results in significantly improved precision and accuracy in the distance domain. Cu^II^-nitrilotriacetic acid (Cu^II^–NTA) spin labeling of double histidine motifs for PD-EPR has been applied successfully to enzymes,^17^ metalloproteins,^18^ and nucleoprotein complexes.^19^

**Figure 1:**
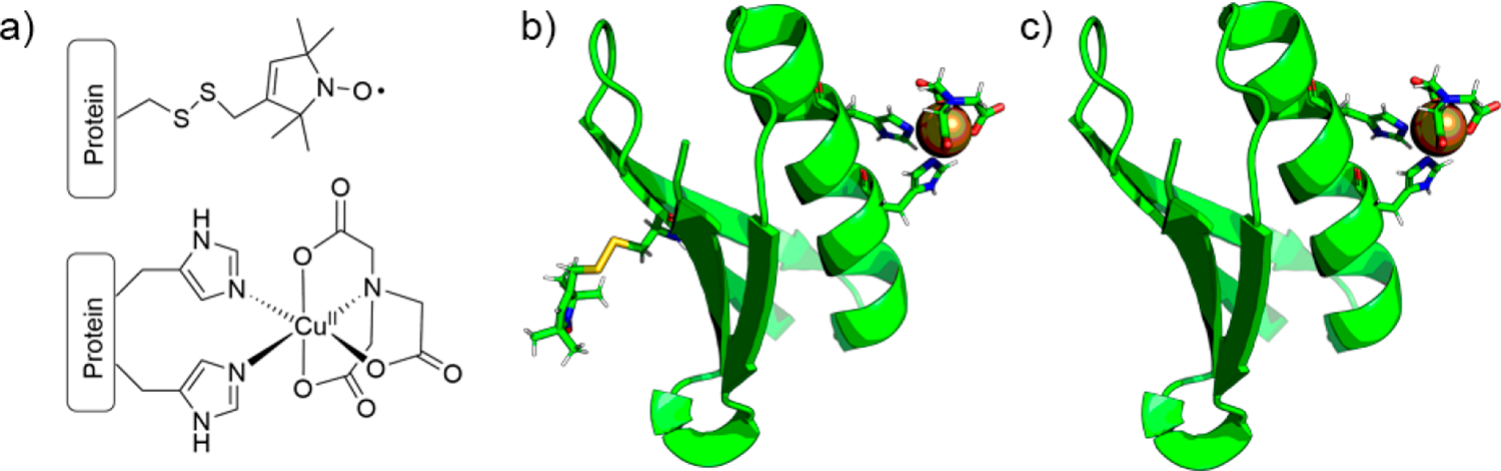
Spin label structures and the GB1 constructs used in this work. (a) MTSL nitroxide conjugated to a cysteine residue, resulting in the R1 side chain (top) and Cu^II^–NTA coordinated to a double-histidine motif (bottom). (b) Cartoon representation of the I6R1/K28H/Q32H GB1 construct, with the R1 nitroxide and Cu^II^–NTA spin labels shown in stick representation. (c) Cartoon representation of the K28H/Q32H GB1 construct, with the Cu^II^–NTA shown in stick representation.

Despite this success, optimization of the spin-labeling approach is nontrivial, because the noncovalency of the interaction predisposes sensitivity to variations in binding affinity, although Cu^II^–NTA labeling may be easier to interpret using molecular dynamics simulations.^20^ For instance, different buffer conditions influence the double-histidine motif labeling efficiency with Cu^II^–NTA.^21^ Furthermore, while the influence of pH upon formation of Cu^II^-chelates has been characterized by CW-EPR previously,^22^ current literature has not shown how pH variations influence binding at the double-histidine motif, particularly under cryogenic temperatures. Similarly, current literature has not addressed competition for double-histidine motif sites by adventitious divalent metal ions, and so warrants investigation. In the current study, *Streptococcus sp.* Group G. protein G, B1 domain (GB1) constructs I6R1/K28H/Q32H (Figure 1b) and K28H/Q32H (Figure 1c) were used as biological model systems, in Cu^II^-nitroxide relaxation-induced dipolar modulation enhancement (RIDME)^23^ pseudotitrations,^10,24^ and isothermal titration calorimetry (ITC) measurements, respectively. Building on our previous work that demonstrated high concentration sensitivity and was reflective of the temperature regime wherein the equilibrium dynamics are frozen-out,^24^ here we establish the use of pulse dipolar EPR for competitive binding assays, and the pH dependence of the equilibrium.

Measurements were first performed in the presence of the model competitor ligand, Zn^II^–NTA, which was chosen because (i) it is a weak ligand for double-histidine motifs compared to Cu^II^–NTA and (ii) it is diamagnetic, so it does not contribute to the detected EPR signal. An EPR silent competitor ligand is desirable because analysis of pseudotitration data is simplified (see Supporting Information (SI) section 1.6). Room temperature ITC data (Figure 2a) fitted to a one-site model where binding stoichiometry could vary indicated a dissociation constant (*K_D_*) of 513 μM. The binding affinity was extrapolated to 235 K (i.e., the temperature at which the binding equilibrium is found to freeze out in our samples, such that diffusional processes cease, meaning our EPR data reflect equilibria at 235 K),^24^ to determine the influence of the competitor ligand upon double-histidine loading efficiency with Cu^II^–NTA under PD-EPR conditions. Importantly, throughout this work the temperature is determined by the self-consistency between EPR and ITC under the assumption that the enthalpy change (Δ*H*) is temperature independent when extrapolating binding affinities to cryogenic temperatures. While the individual assumptions are not necessarily well met, the extrapolation to 235 K using room temperature binding enthalpies is in good agreement with experimental values^24^ even though this might be rooted in a cancellation of errors.

**Figure 2:**
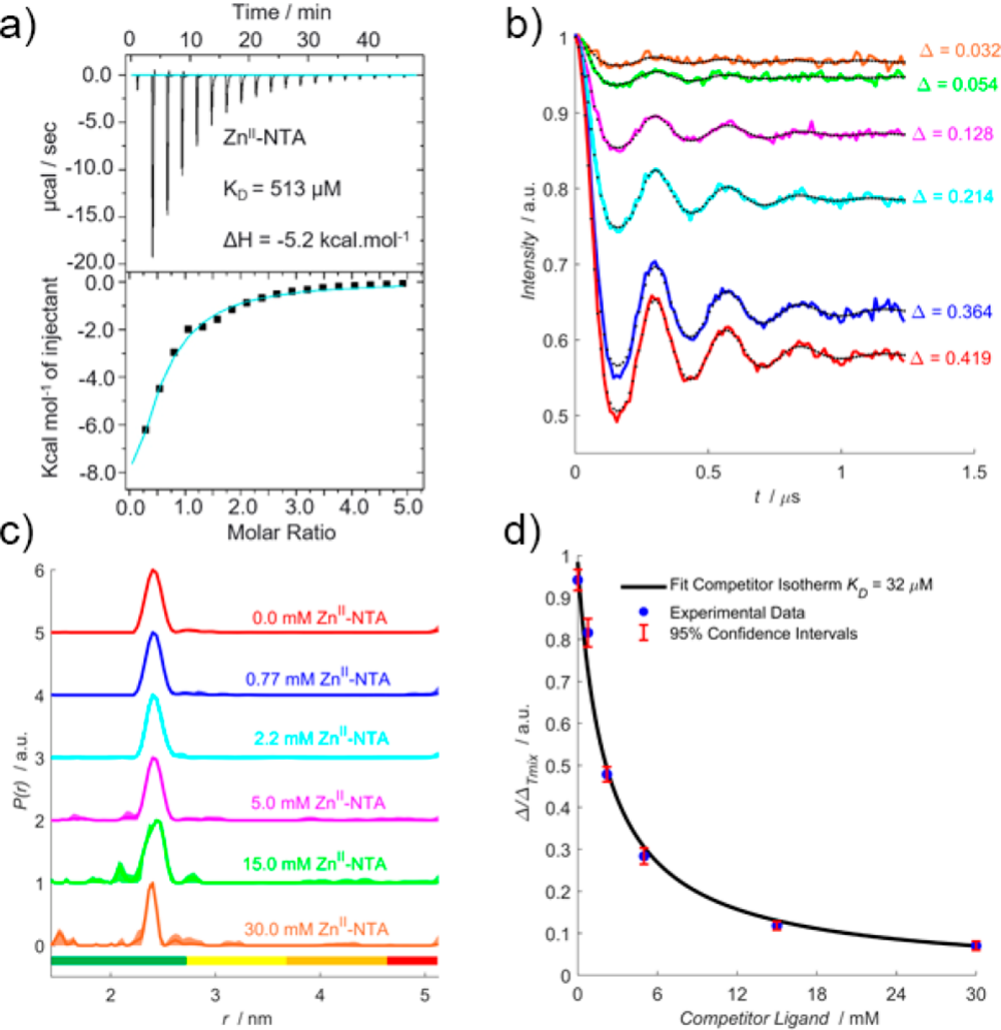
Zn^II^–NTA competitor RIDME pseudotitration. (a) ITC data performed at 298 K, 800 μM 28H/32H GB1 titrated against 12 mM Zn^II^–NTA. (b) RIDME dipolar evolution functions, in absence (red) and presence of 0.77 mM (blue), 2.2 mM (cyan), 5.0 mM (magenta), 15 mM (green), and 30 mM (orange) Zn^II^–NTA, with the corresponding fits shown in dotted black. Modulation depths (Δ) are indicated. (c) Validated RIDME distance distributions, corresponding to the dipolar evolution functions shown in (b). The color scheme is the same in (b) and (c). The concentrations of Zn^II^–NTA are indicated. Color bars represent reliability ranges (green: shape reliable; yellow: mean and width reliable; orange: mean reliable; red: no quantification possible). (d) A univariate fit of the competitor dissociation constant (32 μM) is shown in solid black. Experimental points are shown as the blue scatter, and 95% confidence intervals are shown as the red error bars.

The corresponding RIDME pseudotitration was performed at 1 μM protein concentration in the presence of 10 μM Cu^II^–NTA (to ensure quantitative loading at the double histidine motif prior to addition of competitor ligand (see SI section 1.6)) and varying Zn^II^–NTA concentrations. Importantly, the dipolar evolution functions (Figure 2b) and distance distributions (Figure 2c) show that, in all cases, the expected peak at ∼2.5 nm is retrieved as the only significant feature following data validation. The fitted competitor *K*_D_ value (32 μM) is within 2-fold of that determined from ITC when extrapolated to 235 K (48 μM) (Figure 2d). This suggests that Cu^II^–NTA is robust against the Zn^II^–NTA competitor ligand in vast excess, >1000-fold, even at low μM protein concentrations. Additionally, this benchmarks quantitation of Cu^II^-nitroxide RIDME modulation depths for remotely determining binding affinities of EPR silent ligands, in a competition assay format.

Next, the influence of pH upon double-histidine motif loading efficiency with Cu^II^–NTA was investigated by measuring ITC and RIDME at pH 6.4. Since only deprotonated histidine residues can coordinate Cu^II^–NTA, it follows that binding affinity should decrease under acidic conditions. Indeed, room-temperature ITC performed at pH 5, below the approximate p*K*_A_ of solvent-exposed histidine,^25^ shows negligible binding (see SI section 2.3), and measurements at pH 6.4, fitted to a one-site model, indicated a 20-fold reduction in affinity compared to previous work^24^ (Figure 3a). Extrapolating Δ*H* to 235 K suggested a binding affinity of ∼4 μM.

**Figure 3:**
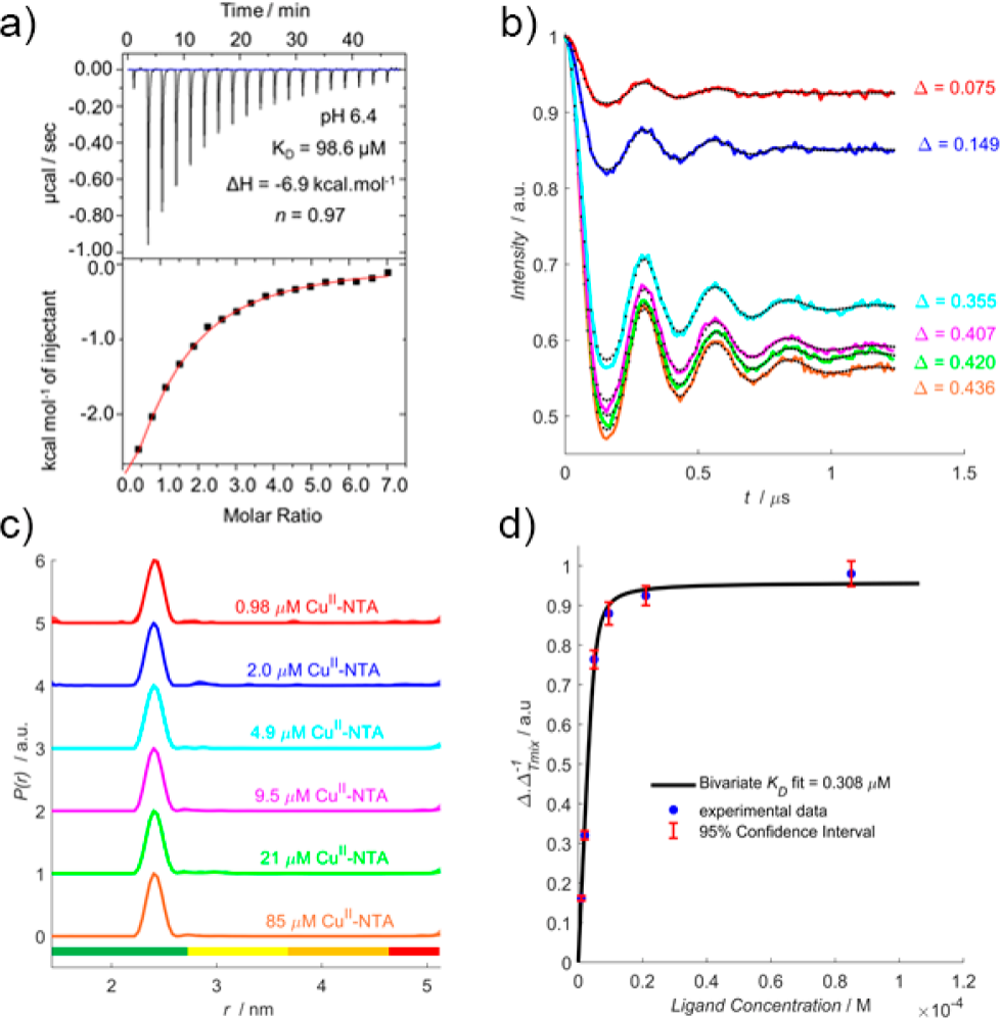
pH 6.4 RIDME pseudotitration. (a) ITC data performed at 298 K, 75 μM K28H/Q32H GB1 titrated against 2 mM Cu^II^–NTA. (b) RIDME dipolar evolution functions, with the corresponding fits shown in dotted black. Modulation depths (Δ) are indicated. (c) Validated RIDME distance distributions, corresponding to the dipolar evolution functions shown in (b). The color scheme is the same in (b) and (c). The concentrations of Cu^II^–NTA are indicated. (d) A bivariate fit of the dissociation constant (0.31 μM) is shown in solid black. Experimental points are shown as the blue scatter, and 95% confidence intervals are shown as the red error bars.

A RIDME pseudotitration was performed at 5 μM protein concentration to validate the room-temperature ITC prediction of reduced affinity under PD-EPR conditions. Significantly, the dipolar evolution functions (Figure 3b) show Cu^II^–NTA binding is only marginally reduced at lower pH, with 1 equiv of Cu^II^–NTA saturating ∼70% of available double-histidine motifs. This is further borne out by the fitted dissociation constant (Figure 3d), 0.31 μM compared to 0.14 μM in previous work at pH 7.4.^24^ The affinity reduced by only 2-fold, indicating that the influence of pH upon double-histidine motif loading may be attenuated at lower temperatures. A possible explanation is that histidine protonation is endothermic,^26^ driving the equilibrium toward the deprotonated state at lower temperatures, compensating for reduced pH and facilitating double-histidine loading. Importantly, this would also imply significantly tighter binding at higher pH, where histidine deprotonation is already favored.

To clarify the disparity between ITC and PD-EPR data at pH 6.4, room-temperature ITC was also performed at pH 8.4 (Figure 4a), fitted to a one-site model, where a 20-fold increase in affinity was predicted (*via* improved thermodynamic favorability of binding) compared to previous work. Another RIDME pseudotitration was performed at 2 μM protein concentration, with dipolar evolution functions (Figure 4b) suggesting moderate improvement in binding affinity. The fitted dissociation constant (Figure 4d) of 0.091 μM indicates the binding affinity is approximately 2-fold higher than at pH 7.4, consistent with observation at pH 6.4 that the influence of pH upon binding affinity is attenuated with decreasing temperature. While an endothermic protonation process would suggest much tighter binding is to be anticipated at pH 8.4, consider that at this pH < 1% of histidine -nitrogen atoms should remain protonated. This may explain why the relative increase in binding affinity is smaller than expected, since the deprotonation is already driven toward completion by the high pH.

**Figure 4:**
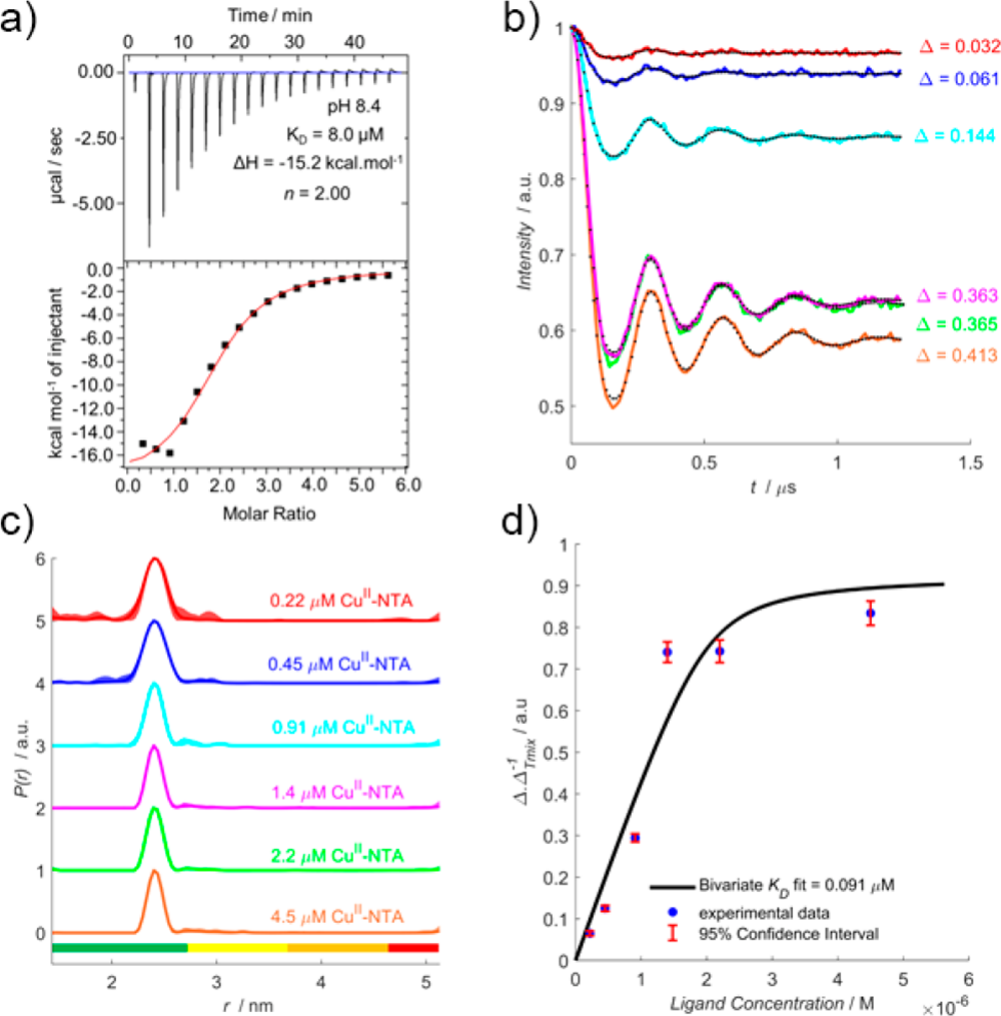
pH 8.4 RIDME pseudotitration. (a) ITC data performed at 298 K, 75 μM K28H/Q32H GB1 titrated against 2.5 mM Cu^II^–NTA. (b) RIDME dipolar evolution functions, with the corresponding fits shown in dotted black. Modulation depths (Δ) are indicated. (c) Validated RIDME distance distributions, corresponding to the dipolar evolution functions shown in (b). The color scheme is the same in (b) and (c). The concentrations of Cu^II^–NTA are indicated. (d) A bivariate fit of the dissociation constant (0.091 μM) shown in solid black. Experimental points are shown as the blue scatter, and 95% confidence intervals are shown as the red error bars.

While the data suggest that spin labeling and measurement at pH 8.4 will afford enhanced loading and sensitivity, it should be noted that the stoichiometry of binding is ∼2, compared to ∼1 at pH 6.4. This may arise from deprotonation of the protein surface that promotes nonspecific binding. This would explain the increased exothermic nature of the binding, if nonspecific or additional binding events contributed to the isotherm and would further inflate the binding affinity when extrapolated to cryogenic temperatures. However, the corresponding distance distributions (Figure 4c) do not contain additional peaks to support this hypothesis.

Perhaps most significantly, these results clearly show that Cu^II^–NTA binding affinity for double-histidine motifs is not strongly perturbed from the high nM concentration regime by fluctuations of pH between 6.4 and 8.4. Coupled with measurements in the presence of competitor ligand Zn^II^–NTA, findings support that Cu^II^–NTA is a highly robust spin label when combined with α-helical double-histidine motifs. This is encouraging for the widespread application of double-histidine motifs in metalloproteins, or in systems where divalent metal cofactors are necessary. Additionally, the benchmarking of a competition assay using PD-EPR is particularly exciting because it allows remote detection of binding interactions with diamagnetic ligands and showcases investigation of competitor ligand binding at significantly reduced material, compared to more established methods like ITC. This will be promising in cases where paramagnetic ligand analogues are not available or cause structural perturbation. PD-EPR also has greater sensitivity than ITC, and the coupling of thermodynamic and structural information allows for the facile monitoring of nonspecific and competitor ligand interactions.^27^ Traditionally, monitoring competitive ligand binding has required expensive radio-labeling and judicious selection of appropriate isotopes.^28,29^ PD-EPR may complement these strategies, while obviating potential cost and safety considerations.

The research data supporting this publication can be accessed at 10.17630/d7138874-55dd-4874-a2e8-c026fbc0b67f.^30^

## Supporting Information Available

(I) Experimental Procedures: Construct Design, Expression and Purification; Pulse EPR Sample Preparation; Metal Chelate Spin Label Preparation; Mass Spectrometry; Pulse EPR Measurements; Competitive Binding Model; Isothermal Titration Calorimetry; UV–visible Spectroscopy. (II) Results and Discussion: Inversion Recovery Measurements; 5-pulse RIDME Measurements; Influence of Differential pH upon Double-Histidine Motif Affinity; Influence of Differential pH upon Cu^II^–NTA Complex Formation; Optimization of Cu^II^–IDA Complex Formation. (III) References. (PDF)
