# Supplementary material for: Pulse Dipolar EPR Reveals Double-Histidine Motif CuII–NTA Spin-Labeling Robustness against Competitor Ions
Source: J Phys Chem Lett. 2021 Mar 13;12(11):2815–9. doi: 10.1021/acs.jpclett.1c00211 (PMC8006131; doi:10.1021/acs.jpclett.1c00211)
Supplement: Supplementary file 1 — jz1c00211_si_001.pdf [file jz1c00211_si_001.pdf]

Pulse Dipolar EPR Reveals Double-Histidine Motif Cu^{II}-NTA Spin-labelling Robustness Against Competitor Ions

Supplementary Material

Joshua L. Wort,[a,b] *Swati Arya,*[b,c] *Katrin Ackermann,*[a,b] *Alan J. Stewart,*[b,c] *Bela E. Bode*[a,b]*

[a]EaStCHEM School of Chemistry, [b]Biomedical Sciences Research Complex, Centre of Magnetic Resonance, University of St Andrews, North Haugh, St Andrews, KY16 9ST, UK.
[c]School of Medicine, University of St Andrews, North Haugh, St Andrews, KY16 9TF, UK

Table of Contents

I	**Experimental Procedures**	S3
1.1	*Construct Design, Expression and Purification*	S3
1.2	*Pulse EPR Sample Preparation*	S3
1.3	*Metal Chelate Spin Label Preparation*	S4
1.4	*Mass Spectrometry*	S4
1.5	*Pulse EPR Measurements*	S4
1.6	*Competitive Binding Model*	S6
1.7	*Isothermal Titration Calorimetry*	S7
1.8	*UV-visible Spectroscopy*	S8
II	**Results and Discussion**	S9
2.1	*Inversion Recovery Measurements*	S9
2.2	*5-pulse RIDME Measurements*	S13
2.3	*Influence of Differential pH upon Double-Histidine Motif Affinity*	S22
2.4	*Influence of Differential pH upon Cu^{II}-NTA Complex Formation*	S24
2.5	*Optimization of Cu^{II}-IDA Complex Formation*	S29
III	**References**	S38

I Experimental Procedures:

1.1 Construct Design, Expression and Purification:

Constructs of *Streptococcus sp.* group G, protein G B1 domain (GB1) were designed, expressed and purified as previously described.[1,2] For completeness the protein sequences for the K28H/Q32H and I6C/K28H/Q32H GB1 constructs are given below in figures S1 and S2, respectively. The positions of the cysteine and double-histidine motif are indicated in cyan and green, respectively.

K28H/Q32H GB1 Protein Sequence:

28 32

MQYKLILNGKTLKGETTTEAVDAATAEHVFKHYANDNGVDGEWTYDDATKTFTVTE

Figure S1. Full amino-acid sequence for the K28H/Q32H GB1 construct used in this work, with each histidine residue of the double-histidine motif shown in green; and residue number indicated above the sequence.

I6C/K28H/Q32H GB1 Protein Sequence:

6 28 32

MQYKLCLNGKTLKGETTTEAVDAATAEHVFKHYANDNGVDGEWTYDDATKTFTVTE

Figure S2. Full amino-acid sequence for the I6C/K28H/Q32H GB1 construct used in this work, with the cysteine residue shown in cyan and each histidine residue of the double-histidine motif shown in green; and residue numbers indicated above the sequence.

1.2 Pulse EPR Sample Preparation:

All material was exchanged into pH-adjusted deuterated buffer A (42.4 mM Na_2HPO_4, 7.6 mM KH_2PO_4, 150 mM NaCl) by first freeze-drying and then re-dissolving in D_2O. For Q-band RIDME samples of 6R1/28H/32H GB1, a total volume of 70 µL was used, with protein concentrations of 1, 2, or 5 µM as stated. All EPR samples were frozen by direct immersion into liquid nitrogen. Zn^{II}-NTA and Cu^{II}-NTA stock solutions were prepared as previously described[1,3]; and for labelling, Zn^{II}-NTA and Cu^{II}-NTA stock solutions with nominal concentrations of 100 and 10 mM were used, respectively.

1.3 *Metal Chelate Spin Label Preparation:*

For preparation of all metal-NTA labels: Cu^{II}-NTA, and Zn^{II}-NTA, , $ZnCl_2$, $CuCl_2$ and NTA were weighed in a glove-box and aliquoted into 1.5 mL reaction tubes. Stock solutions of 100 mM were prepared as previously described.[3] Complete dissolution was ensured by vortexing until solutions were clear of precipitate. For the optimization of Cu^{II}-IDA preparation, solutions of Cu^{II}-IDA spin labels were prepared *via* three distinct methods, i) mixed in a 1:1 equivalence, producing a solution of Cu^{II}-IDA with nominal concentration of 50 mM, before being diluted in milliQ H_2O, ii) mixed in a 1:1 equivalence before dilution with buffer A (150 mM NaCl, 42.4 mM Na_2HPO_4, 7.6 mM KH_2PO_4, pH 7.4), and iii) IDA stock solution was added in 1:8 equivalence with buffer, before addition of 1 equivalent of $CuCl_2$. A dilution series of 20, 10, 5, 2.5 and 1 mM Cu^{II}-IDA was prepared. To simulate spin label loading of protein double-histidine motifs, absorbance spectra were also recorded for the Cu^{II}-IDA dilution series in presence of two equivalents of imidazole. A stock solution of 200 mM imidazole was prepared in buffer A and subsequently diluted upon addition to pre-neutralized 50 mM Cu^{II}-IDA stock solution to 100 mM. Further dilution with buffer yielded the nominal concentrations given above.

1.4 *Mass Spectrometry:*

Mass spectrometry data was collected in-house using a Sciex Matrix Assisted Laser Desorption/Ionization (MALDI) TOF/TOF 4800 mass-spectrometer, with samples crystallized using a matrix of α-cyano-4-hydroxycinnamic acid. I6R1/K28H/Q32H and K28H/Q32H GB1 samples were both prepared at 20 µM concentration in buffer A (42.4 mM Na_2HPO_4, 7.6 mM KH_2PO_4, 150 mM NaCl, pH 7.4), and mass spectra were recorded in the absence of Cu^{II}-chelate.

1.5 *Pulse EPR Measurements:*

All pulse EPR experiments were performed using a Bruker ELEXSYS 580 pulse EPR spectrometer. Temperatures were maintained using a cryogen-free variable temperature cryostat (Cryogenic Ltd) operating in the 1.8-300 K temperature range. All measurements of the electron spin longitudinal relaxation times (T_1) of Cu^{II}-NTA, and all 5-pulse dead-time free RIDME measurements[4] were performed at 30 K, using a high-power 150 W travelling-wave tube (TWT; Applied Systems

Engineering) at Q-band (34 GHz) in a critically coupled 3 mm cylindrical resonator (Bruker ER 5106QT-2w in TE012 mode).

All RIDME measurements were performed as 6-point pseudo-titration series and used the pulse sequence ($\pi/2 - \tau_1 - \pi - (\tau_1 + t) - \pi/2 - T_{mix} - \pi/2 - (\tau_2 - t) - \pi - \tau_2 -$ echo). Detection pulse lengths of 12 and 24 ns ($\pi/2$ and π) and a detection position at the maximum of the nitroxide spectrum. Unless otherwise stated, each trace was acquired using an SRT of 30 ms, a τ_1 of 400 ns, a τ_2 of 1500 ns, with 122 points, 2 shots-per-point, and varying number of scans, as stated. Deuterium ESEEM was suppressed using a 16-step tau-averaging cycle,[5] and unwanted echoes were eliminated using an 8-step phase cycle, for a total of 128 steps per scan. Each measurement was acquired with a short (T_{ref}) and long (T_{mix}) mixing time, of 5 and 200 µs to allow suppression and observation of the dipolar coupling, respectively. Deconvoluted RIDME data was background corrected assuming a stretched exponential background function, with dimension 3-6.[6] Data was processed and validations were performed using DeerAnalysis2018.[7] Background dimension and start-time parameters for data processing were determined by an initial validation, consisting of 56 trials; 8 iterations of background start position (between 5-30% of the total RIDME trace length), and 7 iterations of background dimension (between 3-6 in increments of 0.5). Subsequently, a second round of validations was performed. A total of 896 trials were performed for the second validation round, consisting of 16 white noise iterations (noise level of 1.5), 8 iterations of background start position (between 5-30% of the total RIDME trace length), and 7 iterations of background dimension (between 3-6 in increments of 0.5). These validation trials were also pruned, where trials exceeding the RMSD of the global minimum by $\geq$15% were discarded. Distance distributions are based on the dipolar coupling calculated for the free electron *g*-value and this has not been corrected throughout the manuscript. If the distributions were analyzed and interpreted in detail the x-axes would need to be scaled by a factor 0.938.[8] Furthermore, background imperfections from using a short τ_1 were compensated by dividing by reference traces and did not hamper modulation depth analysis.[9]

All inversion recovery measurements were performed using detection pulse lengths of 16 and 32 ns ($\pi/2$ and π), and an ELDOR π-pulse length of 32 ns. The detection position was consistently placed at the maximum of the Cu^{II}-NTA spectrum. Unless otherwise stated, each trace was acquired using

an SRT of 2 ms, with 2560 points, 25 shots-per-point, and 1-5 scans, as stated. A τ of 800 ns was used, with an approximate time-window length of 500 µs, incremented in steps of 200 ns.

1.6 Competitive Binding Model:

Let us begin by considering a binding equilibrium in presence of a competitor ligand (*A*), with the following two approximations: i) the binding of the ligand (*L*) and (*A*) to a protein (*P*) is mutually exclusive, and ii) the competitor (*A*) binds to (*P*) weakly with respect to (*L*). Here we can define a dissociation constant for both the ligand (K_{DL}) and for the competitor (K_{DA}), given in (S1) and (S2) respectively:

$$K_{DL} = \frac{[P][L]}{[PL]} \tag{S1}$$

$$K_{DA} = \frac{[P][A]}{[PA]} \tag{S2}$$

Rearranging (S1) and (S2) for equilibrium concentrations of protein and protein-competitor complex, given as *[P]* and *[PA]* respectively, yields (S3) and (S4):

$$[P] = \frac{K_{DL}[PL]}{[L]} \tag{S3}$$

$$[PA] = \frac{[P][A]}{K_{DA}} \tag{S4}$$

Here, an expression for total protein, $[P]_0$, can be defined:

$$[P]_0 = [P] + [PL] + [PA] \tag{S5}$$

Substitution of (S3) and (S4) into (S5) yields (S6):

$$[P]_0 = \frac{K_{DL}[PL]}{[L]} + [PL] + \frac{K_{DL}[PL][A]}{[L]K_{DA}} \tag{S6}$$

Symbolic substitution and rearrangement of (S6) to solve for *[PL]* yields (S7):

$$[PL] = \frac{[P]_0[L]}{[L] + K_{DL}\left(1 + \frac{[A]}{K_{DA}}\right)} \tag{S7}$$

Hence the effect of the competitor ligand can be subsumed into an apparent dissociation constant defined as:[10]

$$K_{DLAPP} = K_{DL}\left(1 + \frac{[A]}{K_{DA}}\right) \tag{S8}$$

Owing to assumption ii) (that the competitor ligand A binds to protein P weakly with respect to ligand L), in (S8) we can assume that (S9) is also well met:

$$[A] = [A]_0 \tag{S9}$$

In the simple case of a single ligand-binding site, Cu^{II}-nitroxide RIDME modulation depths (Δ) can be related to the fractional saturation of the protein in terms of total protein and ligand concentrations and ligand dissociation constant K_{DL}, given in (S10):[1]

$$\Delta \times {\Delta_{Tmix}}^{-1} = \frac{[PL]}{[P]_0} = \frac{(K_{DL} + [P]_0 + [L]_0) - \sqrt{(K_{DL} + [P]_0 + [L]_0)^2 - 4[P]_0[L]_0}}{2[P]_0} \tag{S10}$$

where Δ is the observed modulation depth, and Δ_{Tmix} is the asymptotic limit of modulation depth for a given ratio of the mixing time interval T_{mix}, and the longitudinal relaxation time constant T_1 under the mono-exponential approximation, given by (S11):

$$\Delta_{Tmix} = \frac{\left(1 - \exp\left(\frac{-T_{mix}}{T_1}\right)\right)}{2} \tag{S11}$$

Substitution of (S8) and (S9) into (S10) yields (S12):

$$\Delta \times {\Delta_{Tmix}}^{-1} = \frac{\left(K_{DL}\left(1 + \frac{[A]_0}{K_{DA}}\right) + [P]_0 + [L]_0\right) - \sqrt{\left(K_{DL}\left(1 + \frac{[A]_0}{K_{DA}}\right) + [P]_0 + [L]_0\right)^2 - 4[P]_0[L]_0}}{2[P]_0} \tag{S12}$$

This gives an expression for modelling modulation depths as a function of total competitor concentration, in presence of fixed concentrations of protein and non-competitor ligand. For a diamagnetic competitor, $\Delta \times {\Delta_{Tmix}}^{-1}$ is a continuously decreasing function, though this condition is not always well met for a paramagnetic competitor. Therefore, the analysis of the pseudo-titration data is simplified for diamagnetic competitor ligands.

1.7 Isothermal Titration Calorimetry:

All isothermal titration calorimetry experiments used a Malvern MicroCal ITC200 instrument, and were optimized and performed over 19 injections of 2 µL titrant, with an equilibration time of 120

seconds between injections, at 298 K. All solutions were degassed before the use and the final samples were centrifuged immediately before measurements. For the Zn^{II}-NTA measurements, K28H/Q32H GB1 concentration was 800 µM, and titrant concentration was 12 mM. For the variable pH measurements, K28H/Q32H GB1 concentration was 75 µM and titrant concentration was 2.5 mM. Blank conditions of either buffer A (42.4 mM Na_2HPO_4, 7.6 mM KH_2PO_4, 150 mM NaCl, pH 7.4) titrated against addition of 12 mM titrant, or pH adjusted buffer A titrated against addition of 1.0 or 2.0 mM titrant, were recorded for the competitor and variable pH ITC measurements, respectively. Subtraction of the blank measurements from the raw data mitigated the heat of dilution. All data analyses were performed in MicroCal Origin 7 (OriginLab, Northampton, MA) and thermodynamic parameters were derived using standard Marquardt fitting of a single-site binding model, as in our previous work.[1] The fitted equation is given as:

$$Q_{(i)} = \frac{nM_t \Delta H V_0}{2}\left[1 + \frac{X_t}{nM_t} + \frac{1}{nKM_t} - \sqrt{\left(1 + \frac{X_t}{nM_t} + \frac{1}{nKM_t}\right)^2 - \frac{4X_t}{nM_t}}\right] \quad \text{(S13)}$$

Where: $Q_{(i)}$ is the heat content of the solution at the point of the ith injection, K is the binding constant, n is the stoichiometry of binding, ΔH is the molar heat of ligand binding, V_0 is the active cell volume, M_t is the total concentration of macromolecule contained in V_0, X_t is the total concentration of ligand contained in V_0.

1.8 UV-visible Spectroscopy:

All UV-visible absorbance spectra were recorded using a Jenway 6^7 series UV-vis spectrophotometer, in plastic cuvettes with a path length of 10 mm, in single-beam mode. All spectra are recorded at ambient temperature, and with a wavelength resolution of 1 nm, in the wavelength range 320-800 nm. Each sample was blanked before measurement, and all were repeated in triplicate. Data was processed using the Jenway 6^7-series software suite, and were exported to ASCII format for plotting and analysis in Matlab. For quantitation of Cu^{II}-IDA and Cu^{II}-NTA concentration, molar extinction coefficients of 62 $M^{-1} cm^{-1}$ and 59 $M^{-1}cm^{-1}$ at 726 nm, and 800 nm were used, respectively, taken from previous literature.[1,11]

II Results and Discussion:

2.1 Inversion Recovery Measurements:

Inversion recovery measurements were performed to estimate the longitudinal relaxation time of the Cu^{II}-NTA, and the raw data is shown below in figures S3-5, for pH 6.4 and 8.4, and in presence of the competitor Zn^{II}-NTA, respectively. The corresponding mono- and bi-exponential fits are shown as red and blue traces, respectively. The estimates of T_1 fitted under the mono- and bi-exponential approximations, as well as the reciprocal e-times are given in tables S1-3.

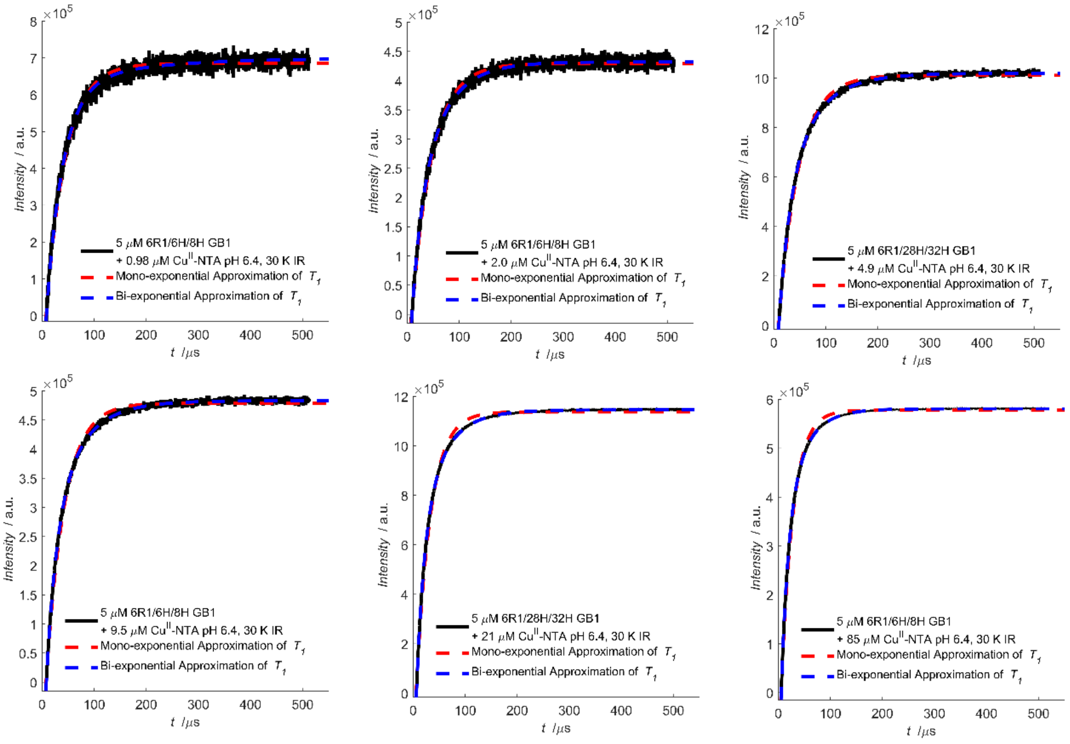

Figure S3. Inversion recovery data at pH 6.4 for 0.98, 2.0 and 4.9 µM Cu^{II}-NTA (top row), and 9.5, 21 and 85 µM Cu^{II}-NTA (bottom row) in presence of 5 µM I6R1/28H/32H GB1 shown left-to-right, respectively. The experimental data is shown in black, with the mono-exponential and bi-exponential fits shown as red and blue dotted lines, respectively.

Sample	Mono-exponential T_1 [μs]	Bi-exponential T_1 [μs]	1/e time [μs]
5 μM 6R1/28H/32H + 0.98 μM Cu^{II}-NTA	37.1 ± 0.34	31.6 (0.91) / 159 (0.09)	44.6
5 μM 6R1/28H/32H + 2.0 μM Cu^{II}-NTA	38.7 ± 0.28	22.1 (0.50) / 55.6 (0.50)	47.0
5 μM 6R1/28H/32H + 4.9 μM Cu^{II}-NTA	38.6 ± 0.19	24.2 (0.61) / 63.9 (0.39)	46.8
5 μM 6R1/28H/32H + 9.5 μM Cu^{II}-NTA	35.0 ± 0.21	21.0 (0.63) / 60.9 (0.37)	42.4
5 μM 6R1/28H/32H + 21.0 μM Cu^{II}-NTA	27.5 ± 0.16	16.2 (0.65) / 49.5 (0.35)	31.6
5 μM 6R1/28H/32H + 85.0 μM Cu^{II}-NTA	22.6 ± 0.12	14.2 (0.67) / 40.9 (0.33)	27.0

Table S1. Mono- and bi-exponential T_1 estimates, and 1/e times for the inversion recovery data shown in figure S3.

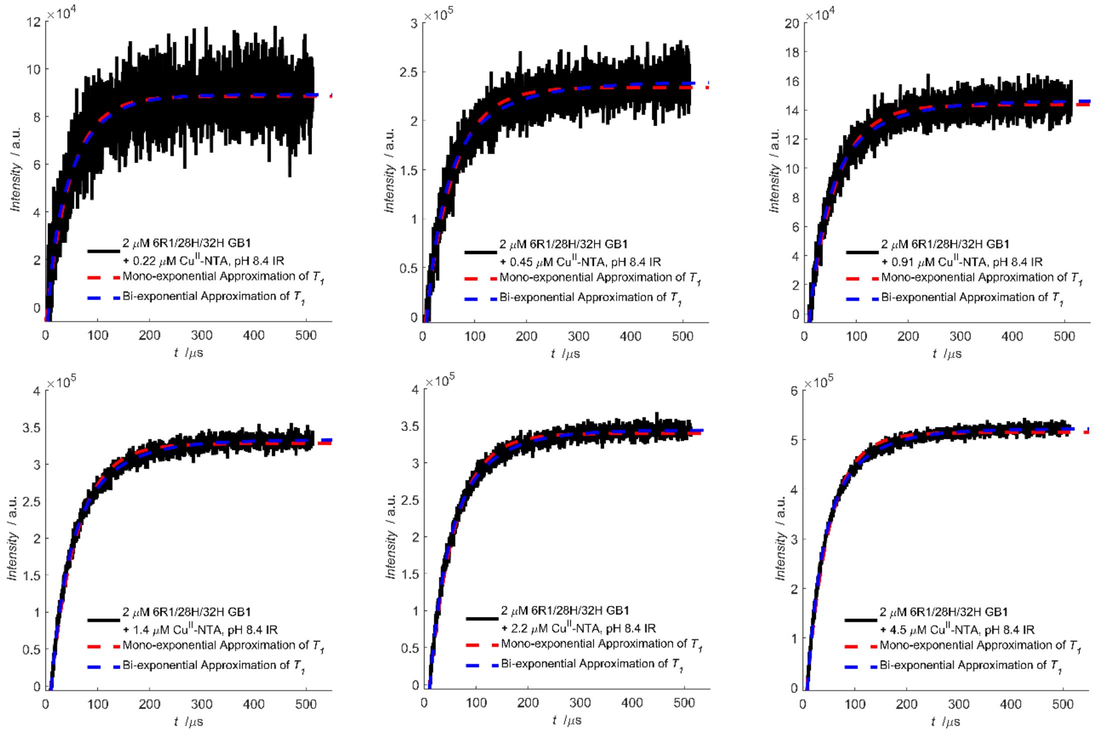

Figure S4. Inversion recovery data at pH 8.4 for 0.22, 0.45 and 0.91 μM Cu^{II}-NTA (top row), and 1.4, 2.2 and 4.5 μM Cu^{II}-NTA (bottom row) in presence of 2 μM I6R1/28H/32H GB1 shown left-to-right, respectively. The experimental data is shown in black, with the mono-exponential and bi-exponential fits shown as red and blue dotted lines, respectively.

Sample	Mono-exponential T_1 [µs]	Bi-exponential T_1 [µs]	1/e time [µs]
2 µM 6R1/28H/32H + 0.22 µM Cu^{II}-NTA	46.6 ± 2.0	9.57 (0.30) / 56.6 (0.70)	41.6
2 µM 6R1/28H/32H + 0.45 µM Cu^{II}-NTA	53.6 ± 1.2	29.7 (0.62) / 104 (0.38)	59.4
2 µM 6R1/28H/32H + 0.91 µM Cu^{II}-NTA	51.9 ± 0.9	29.2 (0.60) / 92.5 (0.40)	56.4
2 µM 6R1/28H/32H + 1.4 µM Cu^{II}-NTA	50.9 ± 0.4	27.8 (0.56) / 82.9 (0.44)	58.4
2 µM 6R1/28H/32H + 2.2 µM Cu^{II}-NTA	49.1 ± 0.4	28.6 (0.58) / 81.0 (0.42)	55.4
2 µM 6R1/28H/32H + 4.5 µM Cu^{II}-NTA	43.2 ± 0.3	24.9 (0.63) / 79.6 (0.37)	48.8

Table S2. Mono- and bi-exponential T_1 estimates, and 1/e time for the inversion recovery data shown in figure S4.

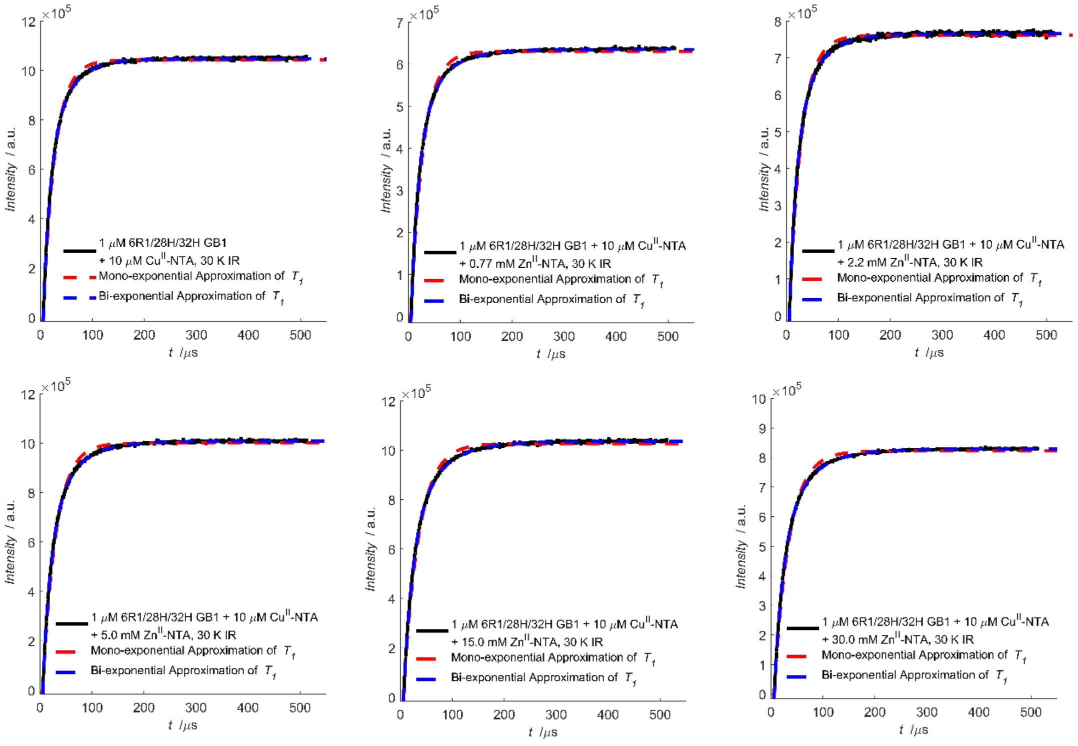

Figure S5. Inversion recovery data for 10 µM Cu^{II}-NTA in presence of 0, 0.77, 2.2 mM Zn^{II}-NTA (top row), and 5.0, 15 and 30 mM Zn^{II}-NTA (bottom row), in presence of 1 µM I6R1/28H/32H GB1 shown left-to-right, respectively. The experimental data is shown in black, with the mono-exponential and bi-exponential fits shown as red and blue dotted lines, respectively.

Sample	Mono-exponential T_1 [µs]	Bi-exponential T_1 [µs]	1/e time [µs]
1 µM 6R1/28H/32H + 0 mM Zn^{II}-NTA	22.7 ± 0.14	13.7 (0.66) / 40.9 (0.34)	27.0
1 µM 6R1/28H/32H + 0.77 mM Zn^{II}-NTA	23.7 ± 0.15	14.8 (0.71) / 48.3 (0.29)	28.0
1 µM 6R1/28H/32H + 2.2 mM Zn^{II}-NTA	23.2 ± 0.14	14.4 (0.68) / 43.3 (0.32)	27.8
1 µM 6R1/28H/32H + 5.0 mM Zn^{II}-NTA	25.7 ± 0.17	15.5 (0.68) / 49.5 (0.32)	29.4
1 µM 6R1/28H/32H + 15 mM Zn^{II}-NTA	29.3 ± 0.19	17.5 (0.67) / 55.5 (0.33)	33.6
1 µM 6R1/28H/32H + 30 mM Zn^{II}-NTA	28.7 ± 0.17	17.3 (0.67) / 53.7 (0.33)	34.2

Table S3. Mono- and bi-exponential T_1 estimates, and 1/e time for the inversion recovery data shown in figure S5.

2.2 5-pulse RIDME Measurements:

RIDME traces recorded with a mixing time interval of 200 µs were deconvoluted with traces recorded with a reference mixing time of 5 µs. Traces and corresponding distance distributions, with shaded regions indicating the ± 2σ confidence intervals are shown below in figures S6-S11, S12-17, and S18-23 for pH 6.4, pH 8.4 and competitor pseudo-titration series, respectively. The color bars represent the reliability ranges described in the DeerAnalysis manual; green indicates shape is reliable, yellow indicates mean and width are reliable, orange indicates mean is reliable, red indicates no quantification is possible. Parameters for the stretched exponential background correction are given in tables S4-6, respectively.

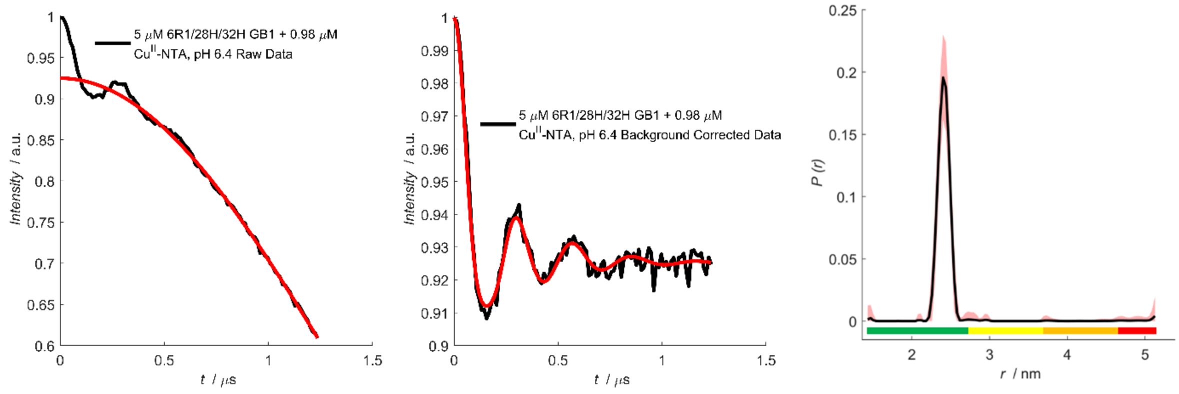

Figure S6: RIDME data of 5 µM 6R1/28H/32H GB1 in presence of 0.98 µM Cu^{II}-NTA. The experimental trace, background corrected data, and distance distribution are shown left-to-right respectively.

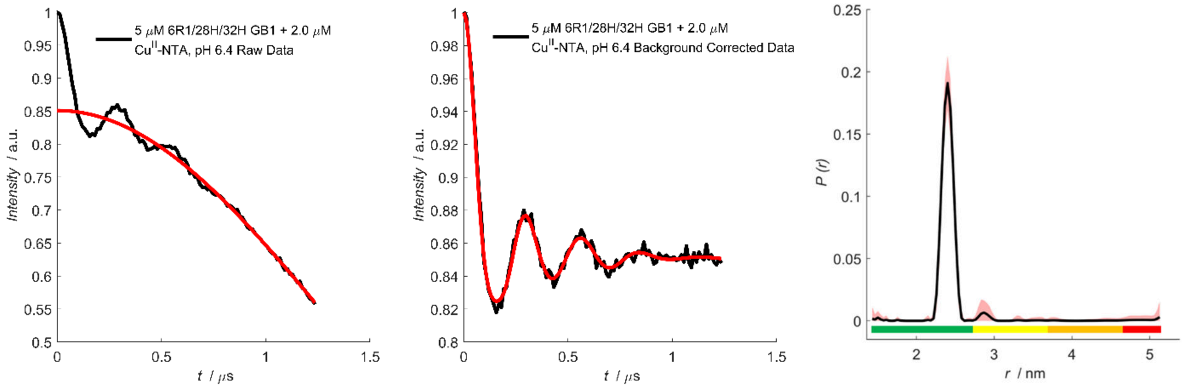

Figure S7: RIDME data of 5 µM 6R1/28H/32H GB1 in presence of 2.0 µM Cu^{II}-NTA. The experimental trace, background corrected data, and distance distribution are shown left-to-right, respectively.

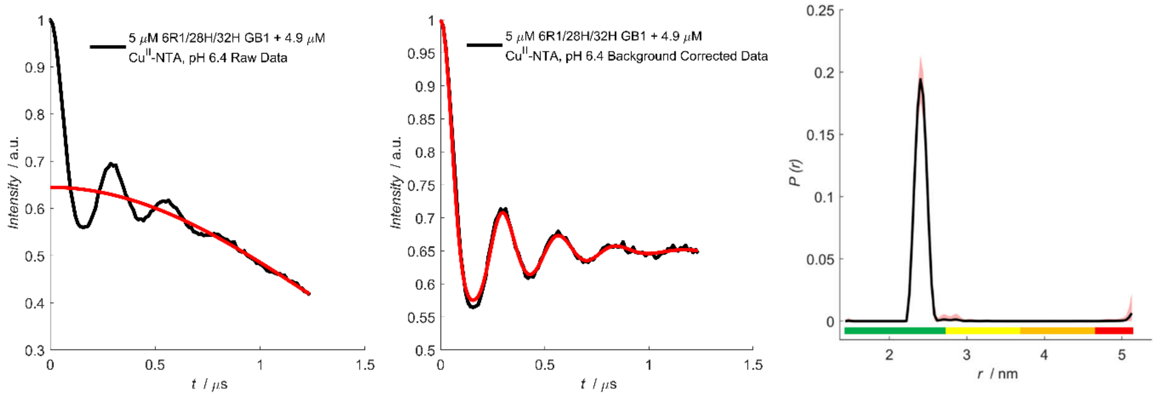

Figure S8: RIDME data of 5 μM 6R1/28H/32H GB1 in presence of 4.9 μM Cu^{II}-NTA. The experimental trace, background corrected data, and distance distribution are shown left-to-right, respectively.

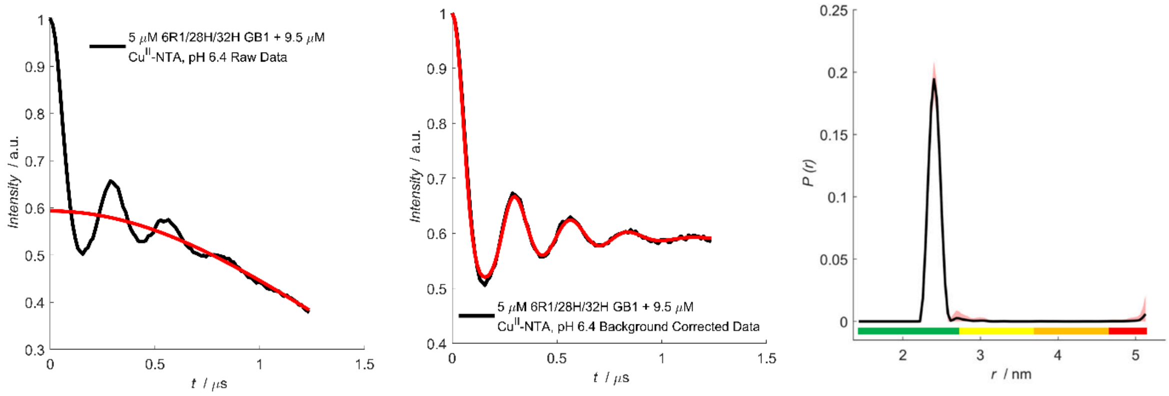

Figure S9: RIDME data of 5 μM 6R1/28H/32H GB1 in presence of 9.5 μM Cu^{II}-NTA. The experimental trace, background corrected data, and distance distribution are shown left-to-right respectively.

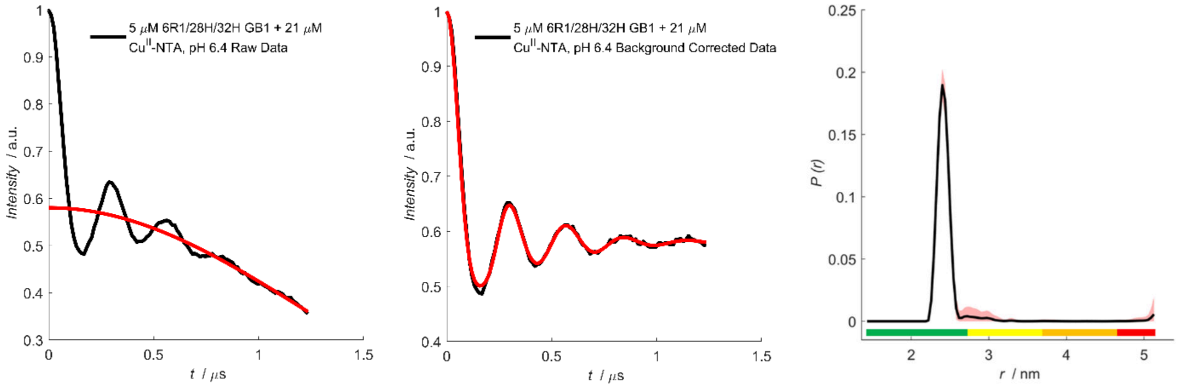

Figure S10: RIDME data of 5 µM 6R1/28H/32H GB1 in presence of 21 µM Cu^{II}-NTA. The experimental trace, background corrected data, and distance distribution are shown left-to-right, respectively.

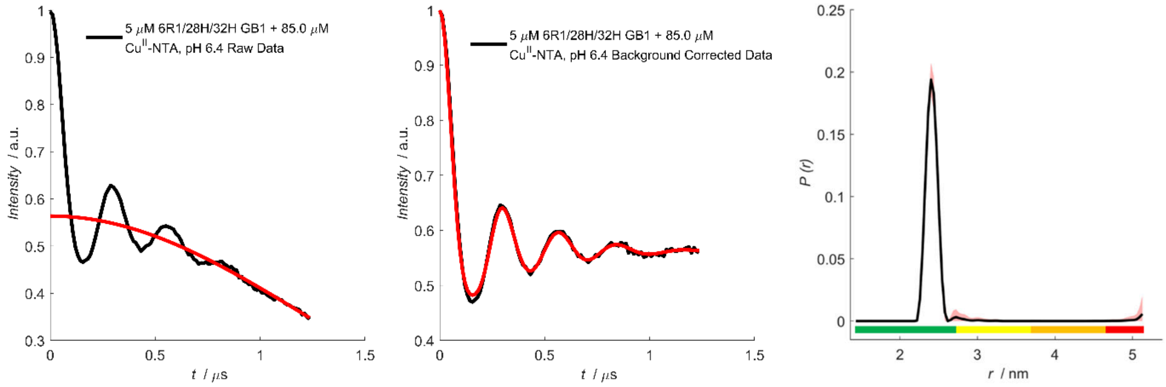

Figure S11: RIDME data of 5 µM 6R1/28H/32H GB1 in presence of 85 µM Cu^{II}-NTA. The experimental trace, background corrected data, and distance distribution are shown left-to-right, respectively.

Sample	Zero-time [ns]	Background Start [ns]	Background Cut-off [ns]	Background Dimension	Modulation depth (Δ)
5 µM 6R1/28H/32H + 0.98 µM Cu^{II}-NTA	206	372	1236	6.00	0.075 ± 3.0 x 10^{-3}
5 µM 6R1/28H/32H + 2.0 µM Cu^{II}-NTA	206	151	1236	6.00	0.149 ± 5.0 x 10^{-3}
5 µM 6R1/28H/32H + 4.9 µM Cu^{II}-NTA	206	62	1236	6.00	0.355 ± 1.1 x 10^{-2}
5 µM 6R1/28H/32H + 9.5 µM Cu^{II}-NTA	206	151	1236	6.00	0.407 ± 1.3 x 10^{-2}
5 µM 6R1/28H/32H + 21 µM Cu^{II}-NTA	205	62	1236	6.00	0.420 ± 1.1 x 10^{-2}
5 µM 6R1/28H/32H + 85 µM Cu^{II}-NTA	205	151	1236	6.00	0.436 ± 1.4 x 10^{-2}

Table S4. Parameters for the stretched exponential background correction and associated modulation depths of the RIDME pseudo-titration shown in figures S6-11.

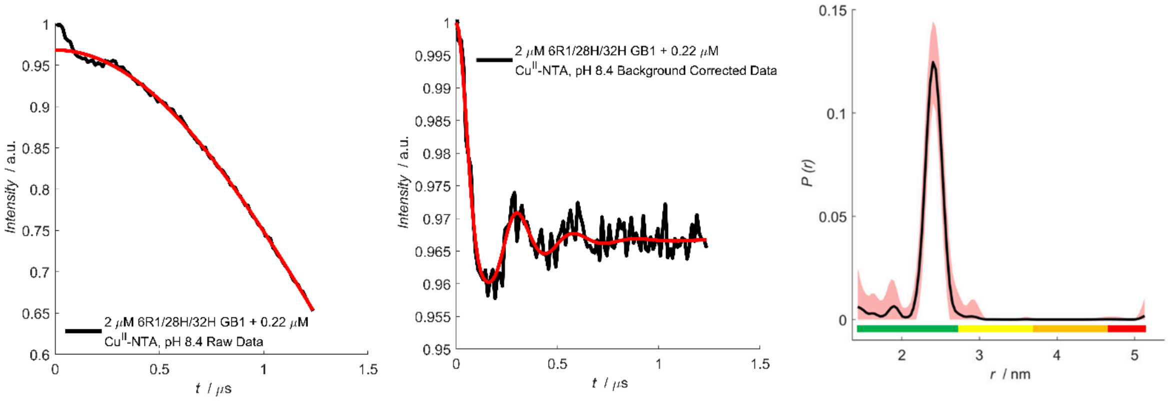

Figure S12: RIDME data of 2 µM 6R1/28H/32H GB1 in presence of 0.22 µM Cu^{II}-NTA. The experimental trace, background corrected data, and distance distribution are shown left-to-right respectively.

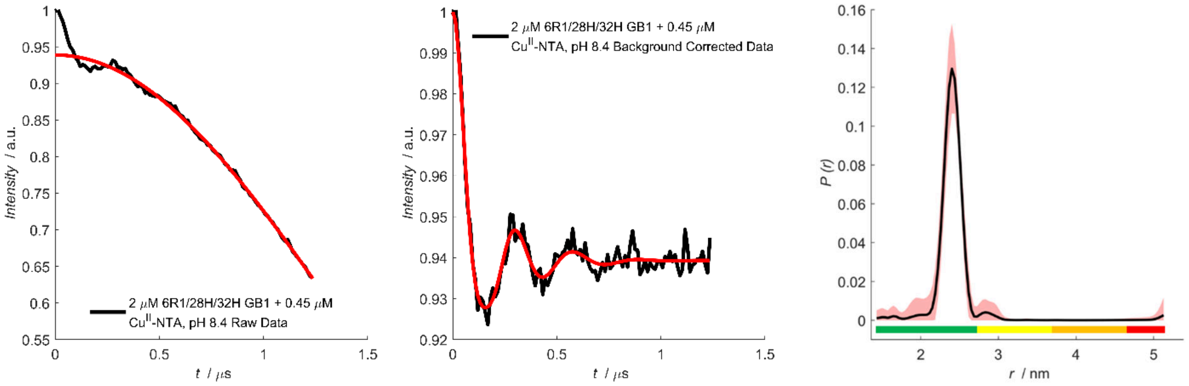

Figure S13: RIDME data of 2 µM 6R1/28H/32H GB1 in presence of 0.45 µM Cu^{II}-NTA. The experimental trace, background corrected data, and distance distribution are shown left-to-right respectively.

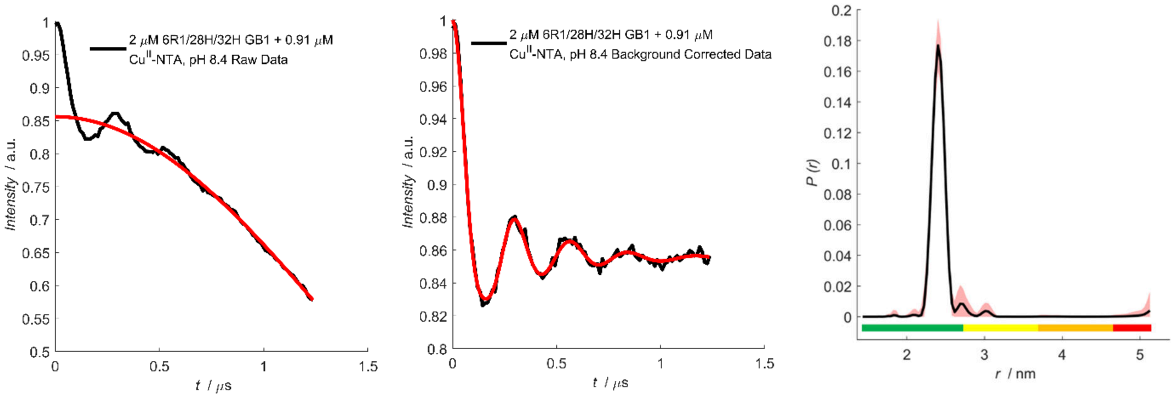

Figure S14: RIDME data of 2 µM 6R1/28H/32H GB1 in presence of 0.91 µM Cu^{II}-NTA. The experimental trace, background corrected data, and distance distribution are shown left-to-right, respectively.

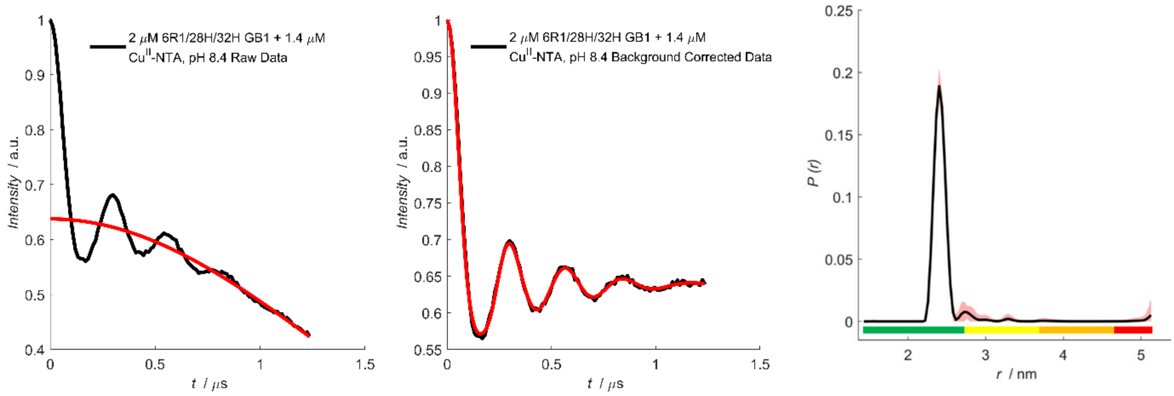

Figure S15: RIDME data of 2 µM 6R1/28H/32H GB1 in presence of 1.4 µM Cu^{II}-NTA. The experimental trace, background corrected data, and distance distribution are shown left-to-right, respectively.

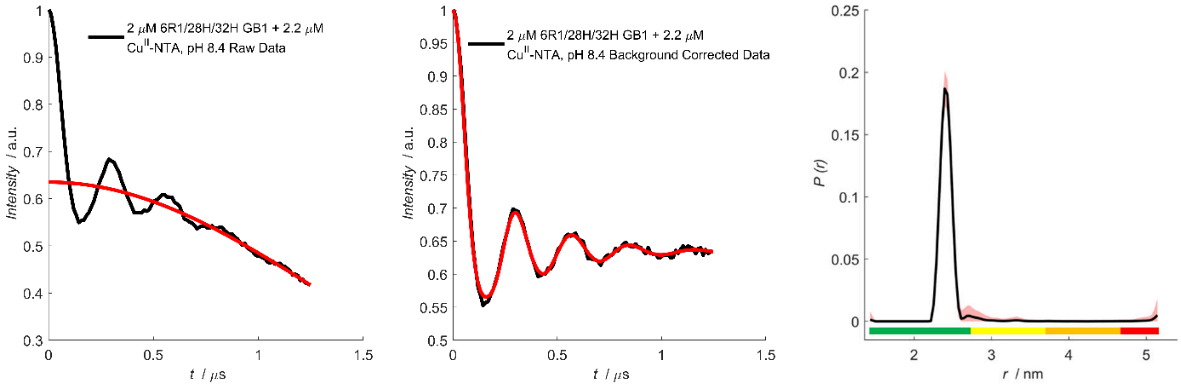

Figure S16: RIDME data of 2 µM 6R1/28H/32H GB1 in presence of 2.2 µM Cu^{II}-NTA. The experimental trace, background corrected data, and distance distribution are shown left-to-right, respectively.

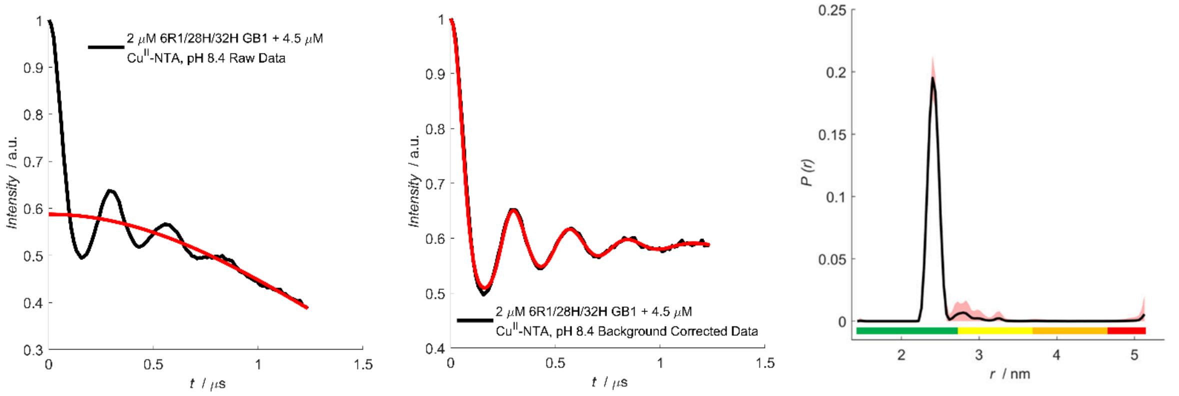

Figure S17: RIDME data of 2 µM 6R1/28H/32H GB1 in presence of 4.5 µM Cu^{II}-NTA. The experimental trace, background corrected data, and distance distribution are shown left-to-right respectively.

Sample	Zero-time [ns]	Background Start [ns]	Background Cut-off [ns]	Background Dimension	Modulation depth (Δ)
2 µM 6R1/28H/32H + 0.22 µM Cu^{II}-NTA	206	194	1236	6.00	$0.032 \pm 2.8 \times 10^{-3}$
2 µM 6R1/28H/32H + 0.45 µM Cu^{II}-NTA	207	328	1236	6.00	$0.061 \pm 3.4 \times 10^{-3}$
2 µM 6R1/28H/32H + 0.91 µM Cu^{II}-NTA	205	151	1236	6.00	$0.144 \pm 4.6 \times 10^{-3}$
2 µM 6R1/28H/32H + 1.4 µM Cu^{II}-NTA	205	62	1236	6.00	$0.363 \pm 1.1 \times 10^{-2}$
2 µM 6R1/28H/32H + 2.2 µM Cu^{II}-NTA	204	62	1248	6.00	$0.365 \pm 1.3 \times 10^{-2}$
2 µM 6R1/28H/32H + 4.5 µM Cu^{II}-NTA	206	62	1236	6.00	$0.413 \pm 1.4 \times 10^{-2}$

Table S5. Parameters for the stretched exponential background correction and associated modulation depths of the RIDME pseudo-titration shown in figures S12-17.

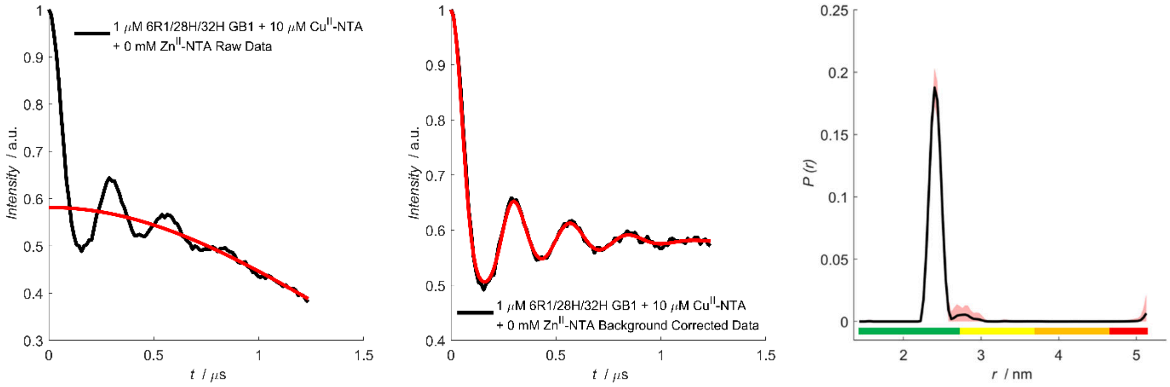

Figure S18: RIDME data of 1 µM 6R1/28H/32H GB1 in presence of 10 µM Cu^{II}-NTA. The experimental trace, background corrected data, and distance distribution are shown left-to-right, respectively.

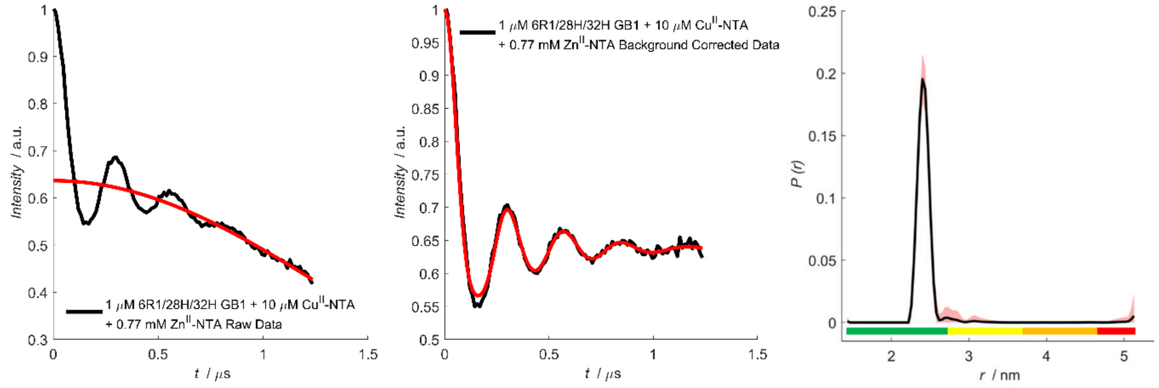

Figure S19: RIDME data of 1 µM 6R1/28H/32H GB1 in presence of 10 µM Cu^{II}-NTA and 0.77 mM Zn^{II}-NTA. The experimental trace, background corrected data, and distance distribution are shown left-to-right, respectively.

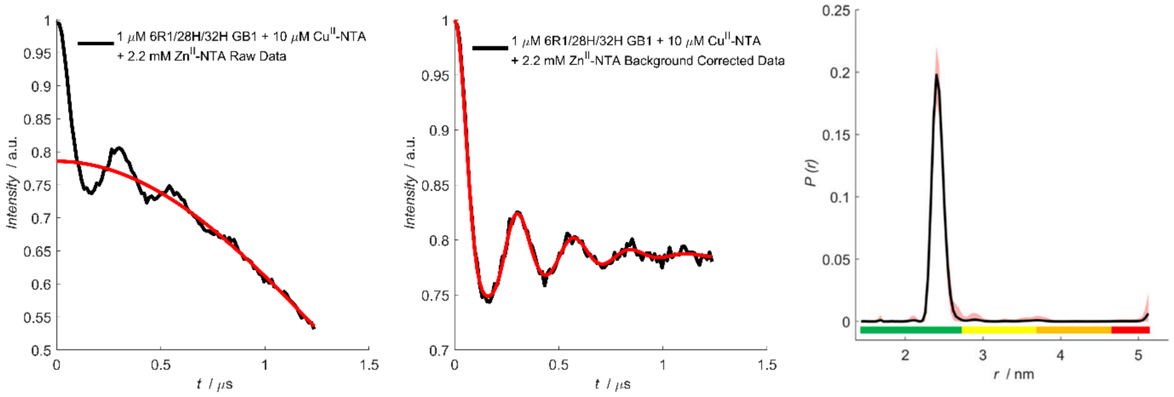

Figure S20: RIDME data of 1 µM 6R1/28H/32H GB1 in presence of 10 µM Cu^{II}-NTA and 2.2 mM Zn^{II}-NTA. The experimental trace, background corrected data, and distance distribution are shown left-to-right, respectively.

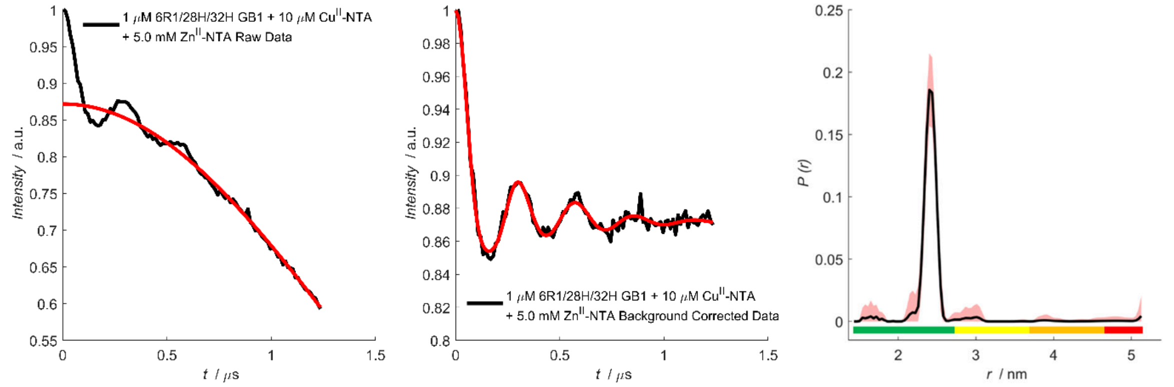

Figure S21: RIDME data of 1 µM 6R1/28H/32H GB1 in presence of 10 µM Cu^{II}-NTA and 5.0 mM Zn^{II}-NTA. The experimental trace, background corrected data, and distance distribution are shown left-to-right, respectively.

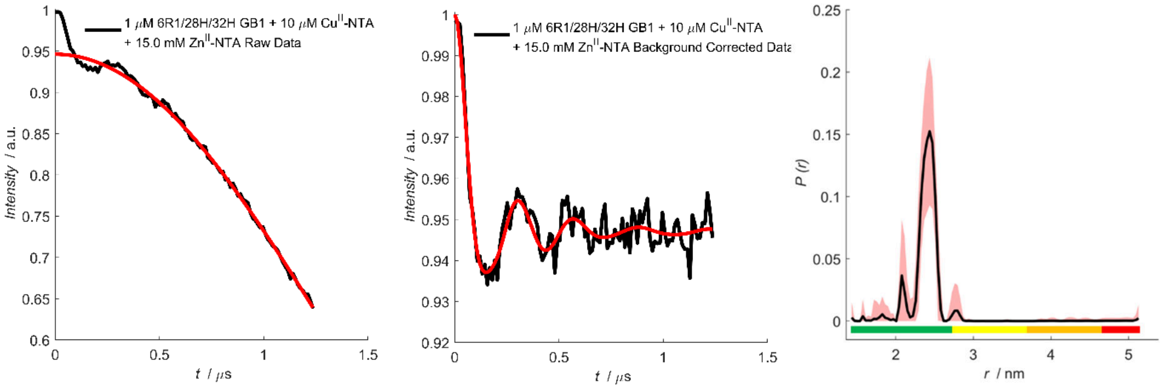

Figure S22: RIDME data of 1 µM 6R1/28H/32H GB1 in presence of 10 µM Cu^{II}-NTA and 15.0 mM Zn^{II}-NTA. The experimental trace, background corrected data, and distance distribution are shown left-to-right, respectively.

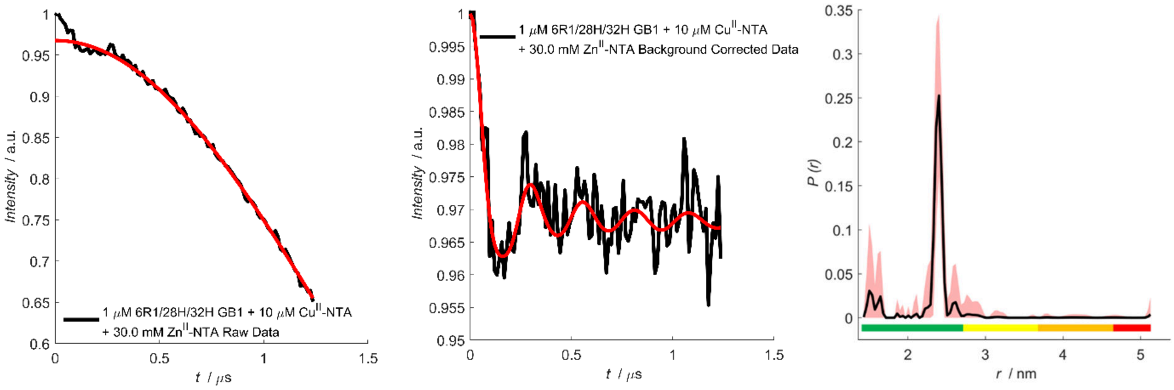

Figure S23: RIDME data of 1 µM 6R1/28H/32H GB1 in presence of 10 µM Cu^{II}-NTA and 30.0 mM Zn^{II}-NTA. The experimental trace, background corrected data, and distance distribution are shown left-to-right, respectively.

Sample	Zero-time [ns]	Background Start [ns]	Background Cut-off [ns]	Background Dimension	Modulation depth (Δ)
1 µM 6R1/28H/32H + 0 mM Zn^{II}-NTA	206	151	1236	6.00	0.419 ± 1.1 x 10^{-2}
1 µM 6R1/28H/32H + 0.77 mM Zn^{II}-NTA	207	328	1236	6.00	0.364 ± 1.5 x 10^{-2}
1 µM 6R1/28H/32H + 2.2 mM Zn^{II}-NTA	205	151	1236	6.00	0.214 ± 8.0 x 10^{-3}
1 µM 6R1/28H/32H + 5.0 mM Zn^{II}-NTA	206	106	1236	6.00	0.128 ± 8.8 x 10^{-3}
1 µM 6R1/28H/32H + 15 mM Zn^{II}-NTA	206	62	1236	6.00	0.054 ± 4.6 x 10^{-3}
1 µM 6R1/28H/32H + 30 mM Zn^{II}-NTA	208	106	1236	6.00	0.032 ± 4.8 x 10^{-3}

Table S6: Parameters for the stretched exponential background correction and associated modulation depths of the RIDME pseudo-titration shown in figures S18-23.

2.3 Influence of Differential pH upon Double-Histidine Motif Affinity:

Spin labelling with Cu^{II}-NTA and double-histidine motifs is a coordination-based approach, therefore it follows that affinity of labelling should be dependent on local pH; if the δ-nitrogen of histidine is protonated it cannot coordinate Cu^{II}-chelates effectively, and so affinity of binding should steeply decrease at pH values below the pK_A of histidine. To test this hypothesis and investigate the influence of pH upon labelling efficiency at double-histidine motifs, isothermal titration calorimetry measurements were performed at pH 5.0 and 9.0, (and pH 6.4 and 8.4 in the main text) with results shown in figures S24 and S25, respectively. Thermodynamic parameters of each measurement are summarized in table S7.

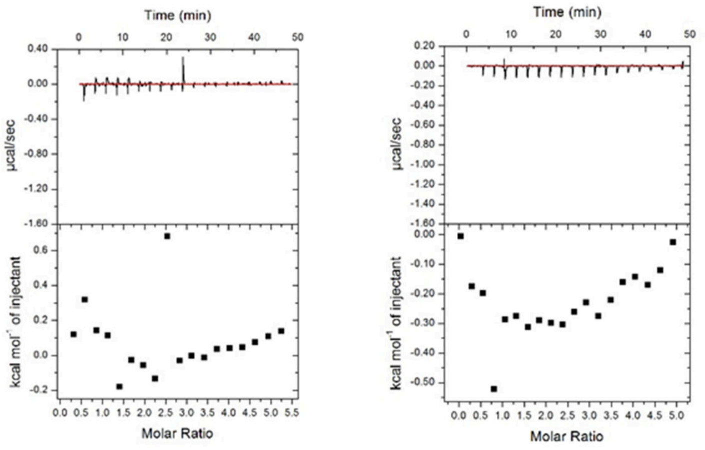

Figure S24: ITC data recorded for 75 µM K28H/Q32H GB1 protein in presence of 2 mM Cu^{II}-NTA at pH 5.0. Repeats are shown in the left and right panels, respectively.

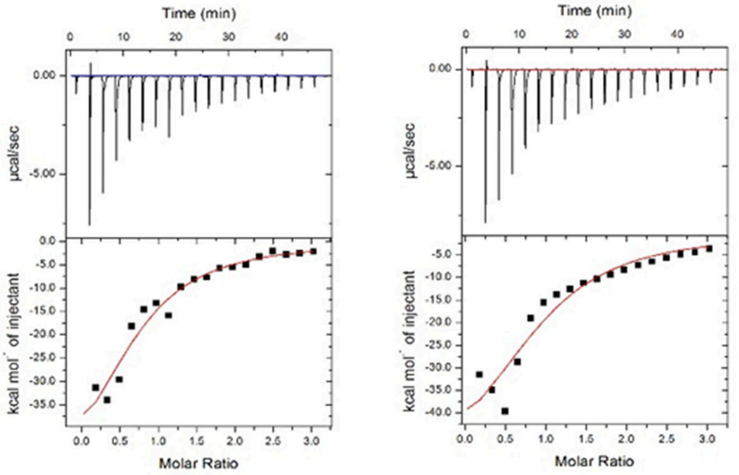

Figure S25: ITC data recorded for 75 µM K28H/Q32H GB1 protein in presence of 1 mM Cu^{II}-NTA at pH 9.0. Repeats are shown in the left and right panels, respectively.

It is seen from figure S24 that at pH 5.0 there is negligible binding at the double histidine motif, which is consistent with the expectation that at pH < 6.0 (the approximate pK_A of histidine), the binding affinity is reduced. This is likely the combination of two effects that preclude coordination of Cu^{II}-NTA at double histidine sites; first complexation of Cu^{II} with the NTA chelator will reduce with lower pH, and similarly, histidine protonation will disrupt coordination by the double histidine. Less Cu^{II}-NTA complex will be available to bind, and fewer histidine residues will be deprotonated and susceptible to coordination at the imidazole δ-nitrogen. At pH 6.4 (figure 3 main text) there is a recovery of double-histidine loading, albeit with a reduced affinity, approximately an order of magnitude weaker binding than previously observed at pH 7.4. This is again in keeping with the expected trend of increasing affinity with increasing histidine deprotonation. Furthermore, it is noted that at pH 8.4 (figure 4 main text), the binding affinity is approximately the same as at pH 7.4, suggesting the histidine residues are already approaching complete deprotonation at pH 7.4. If the pK_A of histidine is taken as 5.5-6.0, then this observation is consistent, since < 1% of histidine residues will be protonated at pH 7.4, and < 0.01% of neighboring histidine pairs will be protonated.

Sample	n	K_D / µM	ΔH / kcal.mol^{-1}	Wiseman factor	Predicted K_D at 235 K / µM
pH 5.0	-	-	-	-	-
pH 6.4	0.97	98.6	-6.94	0.76	4.26
pH 7.4*	1.20	5.00	-7.54	15.0	1.65 x 10^{-1}
pH 8.4	2.04	7.96	-15.2	9.42	8.18 x 10^{-3}
pH 9.0	0.94	25.0	-2.92	3.00	6.67

Table S7: Fit parameters taken from the ITC data shown in figures S24-25 above, and in the main text. (*) indicates that the pH 7.4 ITC measurement was performed using 75 µM 6R1/28H/32H in presence of 2 mM Cu^{II}-NTA ligand.

As can be seen from table S7, there is also an apparent influence of pH on the enthalpy of binding. With increasing pH binding becomes more exothermic, and subsequently affinity increases more steeply with decreasing temperature. This is particularly relevant for pulse EPR applications, since measurements are typically performed at cryogenic temperatures. There is also an increase in the stoichiometry of binding (n) with increasing pH, suggesting a possible role in determining the relative specificity of binding; it should be noted that WT GB1 has been observed to natively bind Cu^{II} *via* coordination to acidic residues, D40 and E56 at the C-terminal domain of the protein.[12] Propensity for this native binding event may increase as acidic residues become deprotonated at higher pH.

2.4 Influence of Differential pH upon Cu^{II}-NTA Complex Formation:

Nominal stock solutions of 100 mM $CuCl_2$ x 6 H_2O and NTA were prepared from aliquots weighed in a glovebox, dissolved in 1 mL pH adjusted milliQ H_2O, (to pH 2.0 or 12.0 respectively), using 2 M HCl and 5 M NaOH. Stock solutions of 10 mM Cu^{II}-NTA were prepared from these stock solutions by being mixed in a 1:1 equivalence before dilution with pH-adjusted buffer A (150 mM NaCl, 42.4 mM Na_2HPO_4, 7.6 mM KH_2PO_4). Respective dilution series were performed at pH 5.0, 6.4, 8.4 and 9.0, and were measured in triplicate, at nominal concentrations of 10, 7.5, 5.0, 2.5 and 1.0 mM Cu^{II}-NTA. Cu^{II}-NTA has an extinction coefficient of 63 $M^{-1}cm^{-1}$ at A_{800nm} at pH 7.4. Spectra are shown for series performed at pH 5.0, 6.4, 8.4 and 9.0 in figures S26-29, respectively. Comparison of the observed A_{800nm} values and those predicted from theory are shown in figure S30. Observed absorbance at 800 nm, and the calculated concentrations are given in tables S8-11.

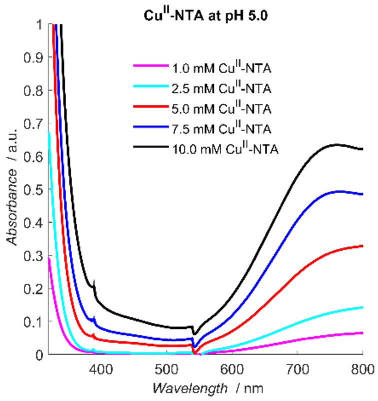

Figure S26. Absorbance spectra recorded for the pH 5.0 Cu^{II}-NTA dilution series, repeated in triplicate, with ± 2σ confidence intervals indicated as the shaded regions. Magenta, cyan, red, blue and black traces correspond to 1, 2.5, 5.0, 7.5, and 10.0 mM Cu^{II}-NTA concentration.

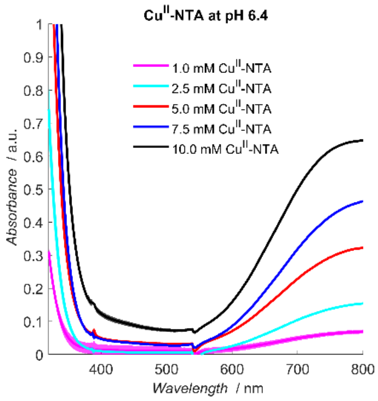

Figure S27. Absorbance spectra recorded for the pH 6.4 Cu^{II}-NTA dilution series, repeated in triplicate, with ± 2σ confidence intervals indicated as the shaded regions. Magenta, cyan, red, blue and black traces correspond to 1, 2.5, 5.0, 7.5, and 10.0 mM Cu^{II}-NTA concentration.

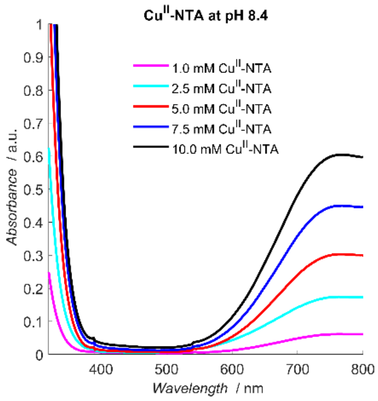

Figure S28. Absorbance spectra recorded for the pH 8.4 Cu^{II}-NTA dilution series, repeated in triplicate, with ± 2σ confidence intervals indicated as the shaded regions. Magenta, cyan, red, blue and black traces correspond to 1, 2.5, 5.0, 7.5, and 10.0 mM Cu^{II}-NTA concentration.

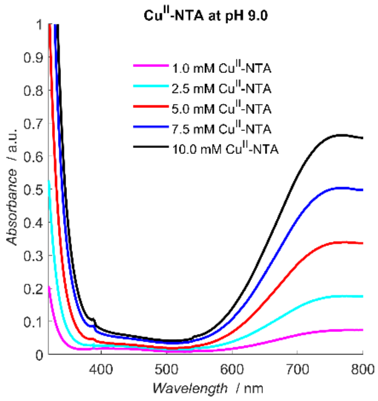

Figure S29. Absorbance spectra recorded for the pH 9.0 Cu^{II}-NTA dilution series, repeated in triplicate, with ± 2σ confidence intervals indicated as the shaded regions. Magenta, cyan, red, blue and black traces correspond to 1, 2.5, 5.0, 7.5, and 10.0 mM Cu^{II}-NTA concentration.

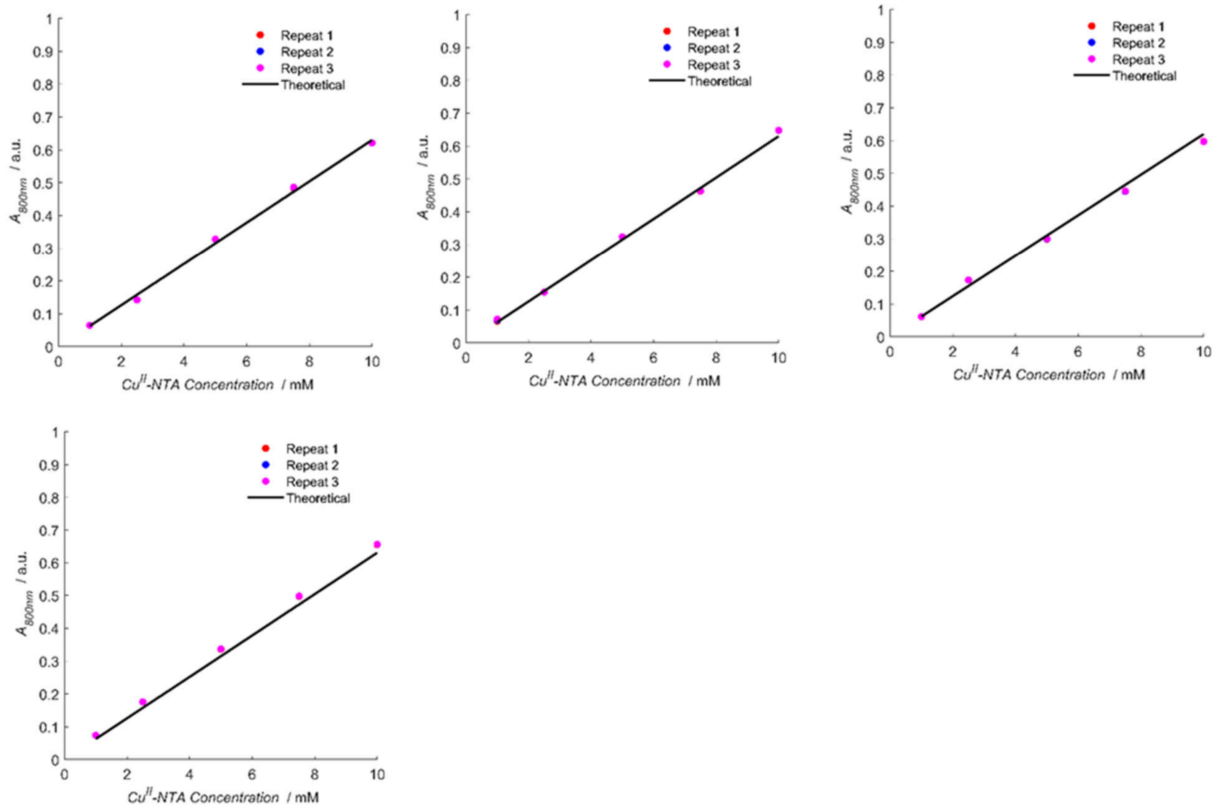

Figure S30. Concentration of Cu^{II}-NTA vs absorbance at 800 nm for pH 5.0, 6.4, 8.4 and pH 9.0, from left-to-right, and top-to-bottom. Experimental data for each repeat set is shown as a red, blue or cyan scatter, with the literature value for the extinction coefficient plotted in black.

Sample	Observed A_{800nm} (a.u.)			Calculated Concentration (mM)		
Repeat	1	2	3	1	2	3
10 mM Cu^{II}-NTA	0.621	0.621	0.621	9.9	9.9	9.9
7.5 mM Cu^{II}-NTA	0.484	0.485	0.486	7.7	7.7	7.7
5.0 mM Cu^{II}-NTA	0.328	0.328	0.328	5.2	5.2	5.2
2.5 mM Cu^{II}-NTA	0.142	0.142	0.142	2.3	2.3	2.3
1.0 mM Cu^{II}-NTA	0.065	0.065	0.065	1.0	1.0	1.0

Table S8. The observed absorbance at 800 nm for the pH 5.0 Cu^{II}-NTA series, taken from the spectra shown in figure S26.

Sample	Observed A_{800nm} (a.u.)			Calculated Concentration (mM)		
Repeat	1	2	3	1	2	3
10 mM Cu^{II}-NTA	0.648	0.648	0.648	10.3	10.3	10.3
7.5 mM Cu^{II}-NTA	0.463	0.464	0.464	7.3	7.4	7.4
5.0 mM Cu^{II}-NTA	0.323	0.323	0.324	5.1	5.1	5.1
2.5 mM Cu^{II}-NTA	0.154	0.154	0.154	2.4	2.4	2.4
1.0 mM Cu^{II}-NTA	0.066	0.069	0.072	1.0	1.1	1.1

Table S9. The observed absorbance at 800 nm for the pH 6.4 Cu^{II}-NTA series, taken from the spectra shown in figure S27.

Sample	Observed A_{800nm} (a.u.)			Calculated Concentration (mM)		
Repeat	1	2	3	1	2	3
10 mM Cu^{II}-NTA	0.598	0.597	0.597	9.5	9.5	9.5
7.5 mM Cu^{II}-NTA	0.445	0.445	0.446	7.1	7.1	7.1
5.0 mM Cu^{II}-NTA	0.299	0.300	0.301	4.7	4.8	4.8
2.5 mM Cu^{II}-NTA	0.173	0.173	0.173	2.7	2.7	2.7
1.0 mM Cu^{II}-NTA	0.061	0.061	0.061	1.0	1.0	1.0

Table S10. The observed absorbance at 800 nm for the pH 8.4 Cu^{II}-NTA series, taken from the spectra shown in figure S28.

Sample	Observed A_{800nm} (a.u.)			Calculated Concentration (mM)		
Repeat	1	2	3	1	2	3
10 mM Cu^{II}-NTA	0.655	0.655	0.656	10.4	10.4	10.4
7.5 mM Cu^{II}-NTA	0.497	0.498	0.498	7.9	7.9	7.9
5.0 mM Cu^{II}-NTA	0.336	0.337	0.337	5.3	5.3	5.3
2.5 mM Cu^{II}-NTA	0.175	0.175	0.176	2.8	2.8	2.8
1.0 mM Cu^{II}-NTA	0.074	0.074	0.074	1.2	1.2	1.2

Table S11. The observed absorbance at 800 nm for the pH 9.0 Cu^{II}-NTA series, taken from the spectra shown in figure S29.

It can be seen from figures S26-29 that the baseline in the region 400-550 nm is not entirely flat, suggesting the presence of precipitate. Qualitatively, precipitate was not observed upon dilution in pH-adjusted buffer for any series, however for the pH 5.0 series, some precipitate was observed at the 1:1 mixing of the acidified $CuCl_2$ and alkalinized NTA stock solutions, suggesting the neutralization reaction was not complete; this may have contributed to the sloping baseline in the region 400-550 nm. From figures S28 and S29 it is seen that the pH 8.4 and 9.0 absorbance series have reasonably flat baselines, and so precipitation does not appear to be problematic, even at alkaline pH. Absorbance at 800 nm seems to be consistently higher for the series recorded at pH

9.0, when compared to pH 5.0, however this does not manifest in a significant shift of the extinction coefficients, and plots in figure S30 show the theoretical absorbance values for an extinction coefficient of 63 $M^{-1}cm^{-1}$. When the absorbance values are linearly fitted, extinction coefficients of 63 and 64 $M^{-1}cm^{-1}$ are found for Cu^{II}-NTA at pH 5.0 and 9.0, respectively.

2.5 Optimization of Cu^{II}-IDA Complex Formation:

Previous literature has indicated that RIDME pseudo-titration modulation depths, particularly for the I6H/N8H/K28R1 series in presence of Cu^{II}-IDA are consistently lower than anticipated.[1] This led to speculation that the equilibrium concentration of Cu^{II}-IDA was also lower than expected, leading to a reduced availability of the chelate to coordinate the double-histidine motif. Interestingly, this effect was not observed with the Cu^{II}-NTA chelate, suggesting the problem does not stem from the protein construct, but rather is specific to the Cu^{II}-IDA label. It is known from literature that the complexation constant of Cu^{II}-NTA is approximately three orders of magnitude greater than for Cu^{II}-IDA,[2] and it has been shown that forming the complex in presence of a tetra-histidine protein can increase PELDOR modulation depth,[2] for otherwise identical experimental conditions. Therefore, to optimize the preparation of Cu^{II}-IDA spin label, UV-visible spectroscopy measurements were performed using three different preparatory conditions, as described in section 1.3.

Results are shown below in figures S31-33 for preparation modes i), ii) and iii). Method i) corresponds to 1:1 mixing of $CuCl_2$ and IDA to produce 50 mM Cu^{II}-IDA, before diluting in milliQ H_2O, method ii) instead dilutes with buffer A (150 mM NaCl, 42.4 mM Na_2HPO_4, 7.6 mM KH_2PO_4, pH 7.4), and method iii) first dilutes the IDA buffer in 1:8 equivalence with buffer A, before addition of 1 equivalent of $CuCl_2$. Methods i), ii) and iii) are discussed subsequently as 'H_2O series', 'neutralized series' and 'buffer series', respectively, to distinguish them. Comparison of the observed A_{726nm} values and those predicted from theory are shown in figure S34. Observed absorbance at 726 nm, and the calculated concentrations are given in tables S12-14 overleaf.

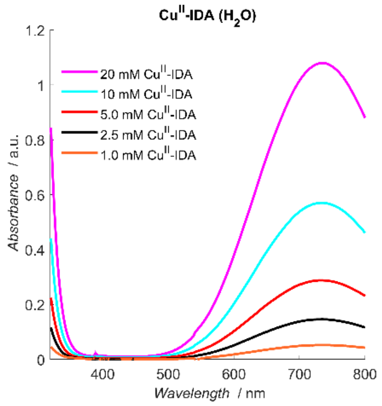

Figure S31. Absorbance spectra recorded for the 'H_2O' Cu^{II}-IDA dilution series (method i), repeated in triplicate, with ± 2σ confidence intervals indicated as the shaded regions. Magenta, cyan, red, black and orange traces correspond to 20, 10, 5, 2.5, and 1.0 mM Cu^{II}-IDA concentration.

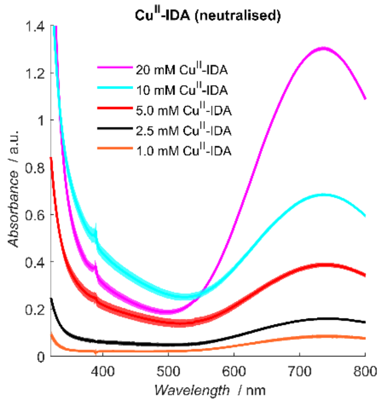

Figure S32. Absorbance spectra recorded for the 'neutralized' Cu^{II}-IDA dilution series (method ii), repeated in triplicate, with ± 2σ confidence intervals indicated as the shaded regions. Magenta, cyan, red, black and orange traces correspond to 20, 10, 5, 2.5, and 1.0 mM Cu^{II}-IDA concentration.

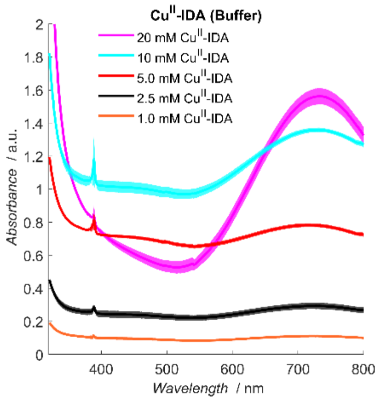

Figure S33. Absorbance spectra recorded for the 'buffer' Cu^{II}-IDA dilution series (method iii), repeated in triplicate, with ± 2σ confidence intervals indicated as the shaded regions. Magenta, cyan, red, black and orange traces correspond to 20, 10, 5, 2.5, and 1.0 mM Cu^{II}-IDA concentration.

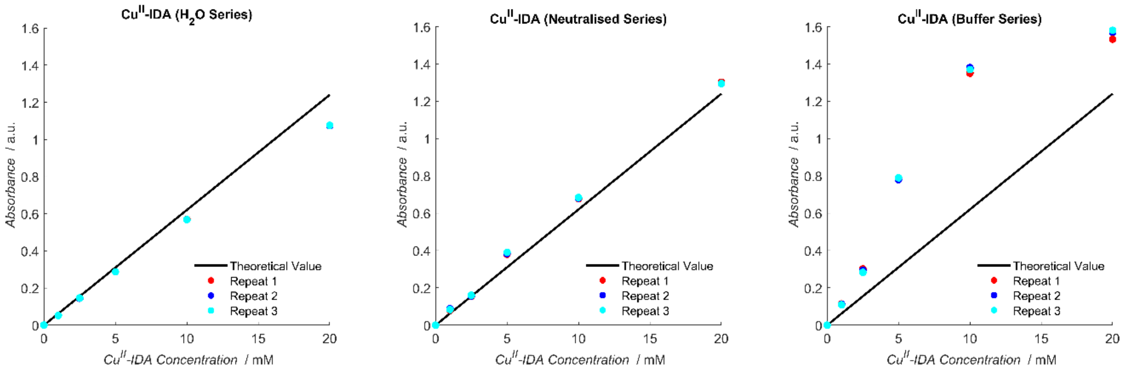

Figure S34. Concentration of Cu^{II}-IDA vs absorbance at 726 nm for the 'H_2O', 'neutralized', and 'buffer' series, from left-to-right. Experimental data for each repeat set is shown as a red, blue or cyan scatter, with the literature value for the extinction coefficient plotted in black. For the 'H_2O' and 'neutralized' series, there is reasonable agreement (within ~20%), with the values predicted from theory.

Sample	Observed A_{726nm} (a.u.)			Calculated Concentration (mM)		
Repeat	1	2	3	1	2	3
20 mM Cu^{II}-IDA	1.075	1.075	1.075	17.3	17.3	17.4
10 mM Cu^{II}-IDA	0.569	0.569	0.568	9.2	9.2	9.2
5.0 mM Cu^{II}-IDA	0.287	0.287	0.287	4.6	4.6	4.6
2.5 mM Cu^{II}-IDA	0.145	0.145	0.145	2.3	2.3	2.4
1.0 mM Cu^{II}-IDA	0.052	0.052	0.052	0.8	0.8	0.8

Table S12. The observed absorbance at 726 nm for each Cu^{II}-IDA solution taken from the spectra shown in figure S31.

Sample	Observed A_{726nm} (a.u.)			Calculated Concentration (mM)		
Repeat	1	2	3	1	2	3
20 mM Cu^{II}-IDA	1.302	1.294	1.295	21.0	20.8	20.9
10 mM Cu^{II}-IDA	0.678	0.681	0.684	10.9	11.0	11.0
5.0 mM Cu^{II}-IDA	0.380	0.384	0.390	6.1	6.2	6.3
2.5 mM Cu^{II}-IDA	0.155	0.157	0.160	2.5	2.5	2.6
1.0 mM Cu^{II}-IDA	0.080	0.088	0.084	1.3	1.4	1.4

Table S13. The observed absorbance at 726 nm for each Cu^{II}-IDA solution taken from the spectra shown in figure S32.

Sample	Observed A_{726nm} (a.u.)			Calculated Concentration (mM)		
Repeat	1	2	3	1	2	3
20 mM Cu^{II}-IDA	1.532	1.570	1.580	24.7	25.3	25.5
10 mM Cu^{II}-IDA	1.352	1.380	1.370	21.8	22.3	22.1
5.0 mM Cu^{II}-IDA	0.780	0.780	0.790	12.6	12.6	12.7
2.5 mM Cu^{II}-IDA	0.301	0.295	0.283	4.9	4.8	4.6
1.0 mM Cu^{II}-IDA	0.113	0.112	0.109	1.8	1.8	1.8

Table S14. The observed absorbance at 726 nm for each Cu^{II}-IDA solution taken from the spectra shown in figure S33.

Figure S31 shows that in the absence of phosphate buffer, the region of the spectrum between 320 and ~500 nm wavelength there is minimal absorbance, regardless of the measured Cu^{II}-IDA concentration. This implies that Cu^{II}-IDA does not contribute to absorbance in this region. In figure S32, the baseline is no longer flat, and shows strong absorbance across a wide range of wavelengths; since the resulting solution is not black, it is possible this is instead the result of Rayleigh scattering, due to precipitation. In figure S33, this effect seems to be further exacerbated, with spectra also lacking the pronounced local maximum at ~750 nm. Instructively, the difference between spectra

in figures S32 and S33 indicates the significance of first allowing the Cu^{II}-IDA complex to form before the addition of phosphate; this is particularly relevant since IDA is not a strong chelator of Cu^{II}, therefore the reaction equilibrium may lie to the left, and result in a population of free Cu^{II}. This likely explains the emergence of the sloping baseline, since a percentage of the free Cu^{II} will precipitate through interaction with the phosphate buffer, or due to alkaline pH. In the case of figure S33, this may be more pronounced, since the IDA chelate is first diluted in buffer, meaning the equilibrium will shift towards the protonated state. Therefore, upon addition of the $CuCl_2$ there will be less IDA available to first form the complex, leaving free Cu^{II}, which can form copper phosphate precipitate.

Measurements were then reproduced in the presence of a 2-fold molar excess of imidazole, to emulate conditions of forming Cu^{II}-IDA chelator in the presence of a double-histidine motif. As above, series are distinguished in discussion as 'H_2O series' and 'buffer series' and spectra are shown respectively in figures S35 and S36. Comparison of the observed A_{726nm} values and those predicted from theory are shown in figure S37. Observed absorbance at 726 nm, and the calculated concentrations are given in tables S15 and S16 overleaf.

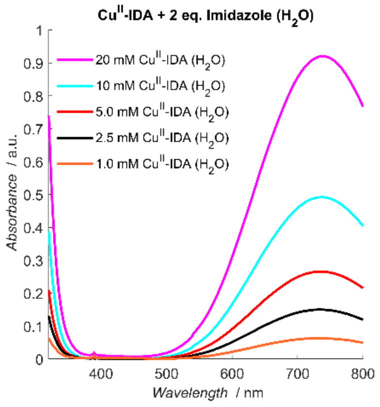

Figure S35. Absorbance spectra recorded for the 'H_2O' Cu^{II}-IDA + imidazole dilution series, repeated in triplicate, with ± 2σ confidence intervals indicated as the shaded regions. Magenta, cyan, red, black and orange traces correspond to 20, 10, 5, 2.5, and 1.0 mM Cu^{II}-IDA concentration (each measured in presence of 2 equivalents of imidazole).

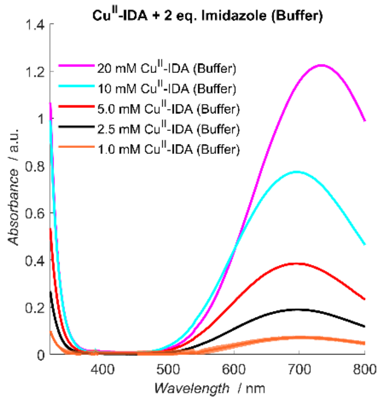

Figure S36. Absorbance spectra recorded for the 'buffer' Cu^{II}-IDA + imidazole dilution series, repeated in triplicate, with ± 2σ confidence intervals indicated as the shaded regions. Magenta, cyan, red, black and orange traces correspond to 20, 10, 5, 2.5, and 1.0 mM Cu^{II}-IDA concentration (each measured in presence of 2 equivalents of imidazole).

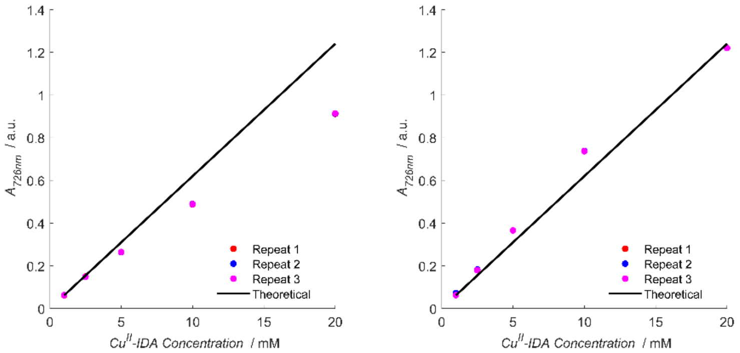

Figure S37. Concentration of Cu^{II}-IDA vs absorbance at 726 nm for the 'H_2O' and 'buffer' series, from left-to-right. Experimental data for each repeat set is shown as a red, blue or magenta scatter, with the literature value for the extinction coefficient plotted in black. For the 'H_2O' series, there is a discrepancy with the values predicted from theory, particularly at higher concentrations.

Sample	Observed A_{726nm} (a.u.)			Calculated Concentration (mM)		
Repeat	1	2	3	1	2	3
20 mM Cu^{II}-IDA	0.912	0.913	0.914	14.7	14.7	14.7
10 mM Cu^{II}-IDA	0.488	0.490	0.489	7.9	7.9	7.9
5.0 mM Cu^{II}-IDA	0.264	0.264	0.265	4.3	4.3	4.3
2.5 mM Cu^{II}-IDA	0.150	0.149	0.149	2.4	2.4	2.4
1.0 mM Cu^{II}-IDA	0.062	0.063	0.063	1.0	1.0	1.0

Table S15. The observed absorbance at 726 nm for each solution taken from the spectra shown in figure S35.

Sample	Observed A_{726nm} (a.u.)			Calculated Concentration (mM)		
Repeat	1	2	3	1	2	3
20 mM Cu^{II}-IDA	1.22	1.22	1.22	19.7	19.7	19.7
10 mM Cu^{II}-IDA	0.737	0.738	0.737	11.9	11.9	11.9
5.0 mM Cu^{II}-IDA	0.366	0.366	0.366	5.9	5.9	5.9
2.5 mM Cu^{II}-IDA	0.183	0.183	0.180*	3.0	3.0	2.9
1.0 mM Cu^{II}-IDA	0.065	0.072*	0.072*	1.0	1.2	1.2

Table S16. The observed absorbance at 726 nm for each solution taken from the spectra shown in figure S36. (*) indicates that the spectra were obtained the following day, using a different blank solution, this may explain the disparity between the repeats.

In presence of an excess of imidazole the region of the spectra between 320 and ~500 nm wavelength is similar to spectra recorded in the absence of phosphate buffer (figure S31). Furthermore, the presence of imidazole appears to completely remove precipitate, suggesting that free Cu^{II} forms adducts with the phosphate buffer. Imidazole co-ordinates both free Cu^{II} and likely stabilizes Cu^{II}-IDA such that the equilibrium lies further to the right, leading to a reduction in the population of free Cu^{II}, as well as reducing the availability of free Cu^{II} to interact with phosphate and precipitate. To ensure that the imidazole did not contribute to absorbance in the visible range, a complete imidazole dilution series was performed, and as seen from figure S38, absorbance is flat in the range 550-800 nm. The λ_{max} value of the imidazole absorbance spectrum occurs at 320 nm, and values are given below in table S17, and plotted as a function of concentration in figure S39.

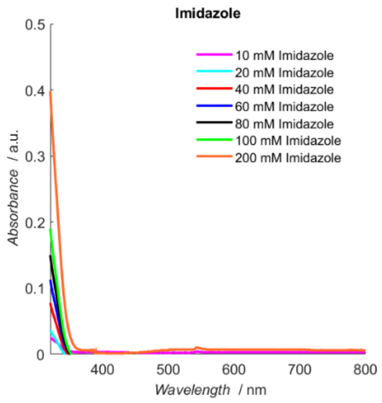

Figure S38. Absorbance spectra recorded for an imidazole dilution series, repeated in triplicate, with ± 2σ confidence intervals indicated as the shaded regions. Magenta, cyan, red, blue, black, red and blue traces correspond to 200, 100, 80, 60, 40, 20, and 10 mM imidazole concentration.

Sample	Observed A_{320nm} (a.u.)		
Repeat	1	2	3
200 mM Imidazole	0.399	0.398	0.397
100 mM Imidazole	0.190	0.190	0.190
80 mM Imidazole	0.150	0.150	0.150
60 mM Imidazole	0.113	0.113	0.112
40 mM Imidazole	0.078	0.078	0.077
20 mM Imidazole	0.037	0.037	0.037
10 mM Imidazole	0.026	0.024	0.024

Table S17. The observed absorbance at 320 nm for each solution taken from the spectra shown in figure S38.

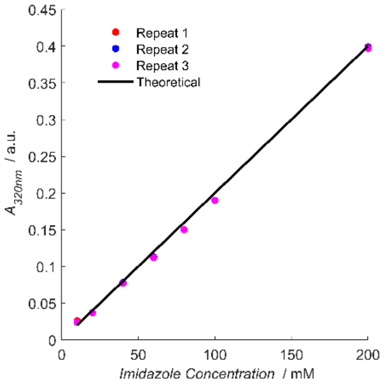

Figure S39. Concentration of Imidazole vs absorbance at 320 nm, the theoretical curve is given using an extinction coefficient of 2 $M^{-1}cm^{-1}$. Experimental data for each repeat set is shown as a red, blue or magenta scatter.

III References:

(1) Wort, J. L.; Ackermann, K.; Giannoulis, A.; Stewart, A. J.; Norman, D. G.; Bode, B. E. Sub-Micromolar Pulse Dipolar EPR Spectroscopy Reveals Increasing Cu^{II}-Labelling of Double-Histidine Motifs with Lower Temperature. *Angew. Chem. Int. Ed.* **2019,** *58*, 11681-111695. https://doi.org/10.1002/anie.201904848

(2) Lawless, M. J.; Ghosh, S.; Cunningham, T. F.; Shimshi, A.; Saxena, S. On the Use of the Cu(II)-Iminodiacetic Acid Complex for Double Histidine Based Distance Measurements by Pulsed ESR. *Phys. Chem. Chem. Phys.* **2017**, *19*, 20959–20967. https://doi.org/10.1039/C7CP02564E

(3) Mehlenbacher, M. R.; Bou-Abdallah, F.; Liu, X. X.; Melman, A. Calorimetric Studies of Ternary Complexes of Ni(II) and Cu(II) Nitrilotriacetic Acid and N-Acetyloligohistidines. *Inorg. Chim. Acta* **2015,** *437*, 152-158. https://doi.org/10.1016/j.ica.2015.08.009

(4) Milikisyants, S.; Scarpelli, F.; Finiguerra, M. G.; Ubbink, M.; Huber, M. A Pulsed EPR Method to Determine Distances between Paramagnetic Centers with Strong Spectral Anisotropy and Radicals: The Dead-Time Free RIDME Sequence. *J. Magn. Reson.* **2009**, *201*, 48–56. https://doi.org/10.1016/j.jmr.2009.08.008

(5) Keller, K.; Doll, A.; Qi, M.; Godt, A.; Jeschke, G.; Yulikov, M. Averaging of Nuclear Modulation Artefacts in RIDME Experiments. *J. Magn. Reson.* **2016,** *272*, 108-113. https://doi.org/10.1016/j.jmr.2016.09.016

(6) Keller, K.; Qi, M.; Gmeiner, C.; Ritsch, I.; Godt, A.; Jeschke, G.; Savitsky, A.; Yulikov, M. Intermolecular Background Decay in RIDME Experiments. *Phys. Chem. Chem. Phys.* **2019,** *21*, 8228-8245. https://doi.org/10.1039/c8cp07815g

(7) Worswick, S. G.; Spencer, J. A.; Jeschke, G.; Kuprov, I. Deep Neural Network Processing of DEER Data. *Sci. Adv.* **2018,** *4*, eaat5218. https://doi.org/10.1126/sciadv.aat5218

(8) Abdullin, D.; Schiemann, O. Pulsed Dipolar EPR Spectroscopy and Metal Ions: Methodology and Biological Applications. *Chempluschem* **2020,** *85*, 353-372.

https://doi.org/10.1002/cplu.201900705

(9) Ritsch, I.; Hintz, H.; Jeschke, G.; Godt, A.; Yulikov, M. Improving the Accuracy of Cu(II)-Nitroxide RIDME in the Presence of Orientation Correlation in Water-Soluble Cu(II)-Nitroxide Rulers. *Phys. Chem. Chem. Phys.* **2019,** *21*, 9810-9830. https://doi.org/10.1039/c8cp06573j

(10) Hulme, E. C.; Trevethick, M. A. Ligand Binding Assays at Equilibrium: Validation and Interpretation. *Br. J. Pharmacol*. **2010**, *161*, 1219-1237. https://doi.org/10.1111/j.1476-5381.2009.00604.x

(11) Gao, J.; Xing, F.; Bai, Y.; Zhu, S. Synthesis, Spectroscopy, and Binding Constants of Ketocatechol-Containing Iminodiacetic Acid and Its Fe(III), Cu(II), and Zn(II) Complexes and Reaction of Cu(II) Complex with H_2O_2 in Aqueous Solution. *Dalt. Trans.* **2014,** *43*, 7964-7978. https://doi.org/10.1039/c4dt00118d

(12) Gamble Jarvi, A.; Cunningham, T. F.; Saxena, S. Efficient Localization of a Native Metal Ion within a Protein by Cu^{2+}-Based EPR Distance Measurements. *Phys. Chem. Chem. Phys.* **2019,** *21*, 10238-10243. https://doi.org/10.1039/C8CP07143H
